# The histone deacetylase inhibitor CT-101 flips the switch to fetal hemoglobin expression in sickle cell disease mice

**DOI:** 10.1371/journal.pone.0323550

**Published:** 2025-05-13

**Authors:** Mayuko Takezaki, Biaoru Li, Hongyan Xu, Nikhil Patel, Rudolf Lucas, Ryan E. Cerbone, Sivanagireddy Koti, Clifford L. Hendrick, Louis H. Junker, Betty S. Pace

**Affiliations:** 1 Department of Pediatrics, Georgia Cancer Center, Augusta University, Augusta, Georgia,; 2 Department of Biostatistics, Data Science and Epidemiology, Augusta University, Augusta, Georgia,; 3 Department of Pathology and Laboratory Medicine, Medical College of Georgia at Augusta University, Augusta, Georgia,; 4 Vascular Biology Center, Department of Pharmacology and Toxicology, Division of Pulmonary and Critical Care Medicine, Medical College of Georgia at Augusta University, Augusta, Georgia,; 5 Cetya Therapeutics, Fort Collins, Colorado,; 6 Colorado State University, Department of Chemistry, Fort Collins, Colorado; Versiti Blood Research Institute, UNITED STATES OF AMERICA

## Abstract

The most common hemoglobin disorder worldwide is sickle cell disease (SCD) caused by a point mutation in the adult β-globin gene. As a result, hemoglobin S production occurs leading to clinical symptoms including vaso-occlusive pain, organ damage, and a shortened lifespan. Hydroxyurea is the only FDA-approved fetal hemoglobin (HbF) inducer in the United States that ameliorates the clinical severity of SCD. Due to challenges with hydroxyurea, our study aimed to address the unmet need for the development of non-chemotherapeutic HbF inducers. We investigated the ability of CT-101, a Class 1 histone deacetylase inhibitor, to flip the γ-globin to β-globin switch in a humanized SCD mouse model. Pharmacokinetic parameters were assessed in CD-1 and Townes SCD mice after a single intraperitoneal drug dose. Similar drug uptake and half-life were observed in both animals. Subsequent studies in β-YAC mice expressing human γ-globin and β-globin genes established the optimal dose of CT-101 that induces HbF without peripheral blood toxicity. Subsequent confirmatory studies were conducted in the SCD mouse treated with intraperitoneal CT-101, demonstrating increases in F-cells, HbF, and γ-globin gene mRNA levels. Hydroxyurea combined with CT-101 significantly decreased spleen size and hemorrhagic infarcts and improved splenic extramedullary hematopoiesis. Our novel agent, CT-101, flipped the switch by activating γ-globin gene transcription and HbF protein synthesis in the preclinical SCD mouse model without significant toxicity in the peripheral blood. These findings support the development of an oral CT-101 formulation for clinical testing in SCD.

## Introduction

Sickle cell disease (SCD) is the most prevalent inherited monogenic blood disorders affecting more than 7 million people worldwide. Approximately 100,000 African Americans live with SCD in the United States, with an average of 2,000 babies born with the disease annually [1]. The cause of SCD is a point mutation in the adult β-globin gene leading to the production of abnormal hemoglobin S which polymerizes under deoxygenated conditions [2]. The net result is sickling of red blood cells (RBCs), a chronic hemolytic anemia, acute painful vaso-occlusive episodes, and progressive tissue and organ damage [3].

Over decades, progress has been made in understanding the pathophysiology and biological complexities of SCD, however the development of specific treatment options has lagged [4]. In 1998, hydroxyurea (HU) was the first US Food and Drug Administration (US FDA)-approved drug for adults with SCD. This repurposed chemotherapeutic agent is the standard of care with recommendations that children and adults with SCD be offered HU treatment starting at 9 months of age [5].

The primary mechanism of action for HU is fetal hemoglobin (HbF) induction triggered by γ-globin gene expression, which is silenced in the first year of life [6]; HU also has anti-inflammatory, and antioxidant effects and serves as a nitric oxide donor [7]. However, HU therapy is myelosuppressive and requires frequent dose monitoring due to neutropenia and thrombocytopenia that can occur. The long-term adverse effects of HU treatment are uncertain, but more recent data suggests there may be a negative impact on growth, fertility, and reproductive capability [8–11].

After decades of research, three new drugs were US FDA-approved to treat vaso-occlusive episodes in people with SCD [12–15] including L-glutamine (Endari), approved in 2017, and crizanlizumab (Adakveo), and voxelotor (Oxbryta) approved in 2019 [16]. These drugs act by mitigating vaso-occlusive pain episodes or improving hemoglobin levels in SCD rather than addressing the fundamental cause of the disease, i.e., hemoglobin S polymerization. Hydroxyurea is the only drug that has been on the market long enough to demonstrate prolonged survival and improved quality of life for individuals living with SCD [8,9]. Although progress has been made, there remains a need to develop better drug candidates for treating SCD that are capable of increasing HbF levels to inhibit hemoglobin S polymerization, reduce RBC sickling and vaso-occlusive episodes, and reduce the clinical severity and mortality associated with SCD.

Various classes of molecules increase HbF expression in tissue culture systems and preclinical animal models [17], however few have made it to clinical trials. Previous studies using CRISPR screens in a human erythroid cell line discovered potential regulators of γ-globin transcription [18] including the nucleosome remodeling and deacetylase complex (NuRD), seven C2H2 zinc finger proteins, and DNA methyltransferase 1. The NuRD complex is comprised of CHD4, MTA2, GATAD2A, MBD2, histone deacetylase 1 (HDAC1) and HDAC2 and two zinc finger proteins, BCL11A and ZBTB7A [19]. DNA methyltransferase 1 silences γ-globin gene transcription by methylating CpG islands in the proximal promoter during development. Exposure to inhibitors such as 5-azacytidine or decitabine mediates hypomethylation of proximal CpG islands to activate γ-globin gene transcription [20]. However, decitabine is currently an intravenous drug formulation and it has antimetabolic and cytotoxic effects when used as an anticancer agent, limiting its use for treating individuals with SCD [20]. An oral formulation of decitabine combined with tetrahydroxyuridine is under investigation as an option for this population [21].

Another major enzyme contained in the NuRD complex are the zinc dependent HDACs, which are divided into different classes based on their structure and intracellular localization. Class I HDACs reside in the nucleus, where they regulate gene expression by removing acetyl groups on histones H3 and H4, creating a closed chromatin structure not conducive to gene transcription. Relevant herein is γ-globin gene silencing by Class I HDAC1 and HDAC2 [6]. At birth, HbF levels are high, which inhibits hemoglobin S polymerization to mitigate clinical symptoms. However, a genetic switch from fetal γ-globin to adult β^S^-globin gene transcription occurs in the first year of life in SCD leading to high hemoglobin S levels and overt clinical symptoms. The switch is mediated by the repressors HDAC1, HDAC2, MBD2, BCL11A, and arginine methyltransferase PRMT5, which bind in the γ-globin gene promoter [6]. Thus, the inhibition of any one of these proteins should theoretically be beneficial for HbF activation and treating individuals with SCD.

Histone deacetylase inhibitors such as butyrate [22] have been evaluated in clinical trials. However, this first-generation pan-cellular HDAC inhibitor required parenteral treatment and produced intolerable myelosuppression, limiting its development for SCD therapy. Vorinostat is a second pan-cellular HDAC inhibitor evaluated in a phase 1/2 clinical trial which did not induce HbF expression in adults with SCD. The authors propose the use of HDAC1- or HDAC2-specific inhibitors to avoid anticipated systemic toxicities previously documented with pan-cellular inhibitors in humans [23]. Thus, the development of Class 1 HDAC inhibitors as a novel therapy for individuals with SCD is supported by molecular mechanistic evidence [24].

We previously conducted *in vitro* studies using primary erythroid progenitors derived from adults with SCD to develop a novel Class I HDAC inhibitor, CT-101 [25]. Treatment with CT-101 was not toxic to erythroid cells with normal growth and maturation, and induction of HbF expression; moreover, an additive effect was observed when CT-101 was combined with HU. To expand on these findings, herein we evaluated CT-101 in two preclinical animal models including the humanized β-YAC and Townes SCD mouse models. Once the optimal CT-101 dose that induces HbF was established in β-YAC mice, we demonstrated the ability of CT-101 to preferentially activate γ-globin and repress β^S^-globin transcription in SCD mice. Moreover, CT-101 treatment did not cause significant adverse effects on peripheral blood cell counts. The results of our preclinical studies and implications for developing a novel HbF inducer for treating SCD are discussed.

## Materials and methods

### Synthesis of CT-101

The proprietary prodrug CT-101, free thiol bioactive form CT-101S and CT-101 dimer (Table 1) were designed in the laboratories of Cetya Therapeutics, Inc. (Fort Collins, CO). CT-101 was synthesized from 3 critical building blocks of starting materials (“fragments”) including α-methyl cysteine, peptide base and thiazole-thiazoline fragments. Recently we improved the synthetic route for the macrocycle prodrug which is now synthesized in four (4) steps with an overall yield of 30%. Additional benefits of the improved synthetic route include eliminating a high potency pharmacophore and the need for containment to protect manufacturing personnel (US patent # 12227522) [26]. The prodrug purity was characterized by HPLC, and identity by NMR and optical rotation. A single 1.1 g lot of CT-101 was used to develop the LC-MS bioanalytical method and for drug dosing in animals. The purity of the CT-101 was 99.0% by HPLC at time zero, 98.7% after one year, and 98.9% after two years when stored at -20^o^C under a blanket of argon.

**Table 1:** Structure of the CT-101 Prodrug and Free Thiol, and Dimer Forms.

Compound	CT-101	CT-101S	CT-101 Dimer
CT-101 Analogue	Parent Prodrug Form	Free Thiol Bioactive Form	Dimer Form
Chemical Formula	C_29_H_43_N_5_O_4_S_3_	C_21_H_29_N_5_O_3_S_3_	C_42_H_56_N_10_O_6_S_6_
Molecular Weight	621.9	495.7	991.4

### Bioavailability and pharmacokinetic studies in animals

All formulation candidates for intraperitoneal (IP) administration and assessment of bioavailability and pharmacokinetic (PK) parameters in animals were prepared onsite at the Contract Research Organization Inotiv (Lafayette, IN). Formulation components varied in the four formulation candidates, and all were acceptable for human use. Further, bench scale testing demonstrated the stability of the four CT-101 formulations at room temperature for up to 4 hours. To assess bioavailability, animals were given a single IP dose of CT-101, blood collected by tail vein into EDTA K3 treated tubes. The samples were centrifuged under refrigerated conditions at 5000–8000 RPM and processed to collect plasma in 0.5 mL microtube and were frozen at -70^o^C until transferred to Inotiv (St. Louis, MO) for bioanalysis. Plasma samples were prepared at time points up to 8 or 24 hours to determine CT-101S and CT-101 dimer levels using Phoenix WinNonlin software (Version 8.1 or higher) non-compartmental analysis (linear trapezoidal rule for area under the curve [AUC] calculations). Nominal dose values and sampling times were used for calculations. Any concentrations reported as BLQ (below limit of quantitation) at a threshold of 1.00 ng/mL CT-101S and 10.0 ng/mL CT-101 dimer, were set to zero. For calculations of AUC on study day 1, the plasma levels at time zero for CT-101S and CT-101 dimer were set to zero. Pharmacokinetics analysis included the determination of Cmax (maximum concentration), T_max_ (time of maximum concentration), T_last_ (final time point), and T_1/2_ (drug half-life). The mean CT-101S and CT-101 dimer, plasma concentrations and CV% (coefficient of variation percentage) were presented as individual and mean values.

### Quantitation of CT-101 analogs in plasma

The whole blood was collected from all animal models in EDTA-K3 treated tubes and processed within 30 min to collect plasma, stored at -70^o^C until bioanalysis by Inotiv. They previously developed a LC-MS method using a Sciex ExionLC UPLC and AB Sciex API 4000 triple-quad MS to quantify total CT-101 as the free thiol form (CT-101S) following the addition of the reducing agent dithiothreitol and an internal standard tolbutamide to account for extraction efficiency. CT-101 dimer was determined in the same way but without dithiothreitol. Paired plasma samples from animal studies were analyzed with and without dithiothreitol. Tolbutamide was added to both samples extracted with acetonitrile, and CT-101S levels were determined in the reduced sample and CT-101 dimer determined in the non-reduced sample. The method was shown to be fit-for-purpose with a linear response from 1–1,000 ng/mL for CT-101S and 10–5,000 ng/mL for CT-101 dimer.

### CT-101 treatments in β-YAC and Townes SCD transgenic mice

The β-YAC transgenic mouse model contains the full-length 81 kb human *HBB* locus, including the locus control region. The five functional human globin genes “5-ε-^G^γ-^A^γ-δ-β-3” on chromosome 11 in the *HBB* locus undergo normal developmental regulation after birth with γ-globin gene silencing and adult β-globin gene activation, a process known as hemoglobin switching [27]. Study 1 was conducted in β-YAC mice (5–6 months old) treated with CT-101 at two doses (5 and 10 mg/kg) administered once daily by intraperitoneal injections (IP), three times a week (M, W, F) suspended in FORM-1, and HU (100 mg/kg) administered once daily for five days per week (M-F) for four weeks by IP injections. FORM-1 (vehicle for CT-101) and water (vehicle for HU) controls were included in the study design. Each treatment group consisted of six mice per group (3 males and 3 females), and data was combined for 2 replicates for statistical analysis (n = 12). We collected tail vein blood at weeks 0, 2, and 4 and analyzed complete blood counts and differentials using an automated Micros 60 device (HORIBA Medical/ABX Diagnostics, Irvine, CA). The level of F-cells and mean fluorescence intensity (MFI) was determined by flow cytometry as previously published [28]. In Study 2, CT-101 (10 mg/kg) alone or combined with HU (100 mg/kg) was evaluated over a 4-week treatment period to determine additive effects.

Study 3 confirmed HbF induction in the Townes SCD mouse model, which completes hemoglobin switching from human γ-globin to β^S^-globin shortly after birth [29]. These mice display the same clinical symptoms as people with SCD, including erythrocyte sickling, hemolysis and anemia, splenomegaly, and vaso-occlusive episodes [30]. The study design was the same as in Studies 1 and 2, using SCD mice (4–6 months old) dosed once daily by IP injections, with water, HU (100 mg/kg), vehicle (FORM 3), CT-101 in FORM-3 (10 mg/kg), and combined HU and CT-101 administration. The study period was 4 weeks, and two independent replicates were combined for statistical analysis (n = 8–12). Blood collected at weeks 0, 2, and 4 were analyzed for complete blood count and differential, and F-cells and MFI quantified by flow cytometry. Blood stained with acridine orange (BD retic-count reagent) quantified reticulocytes by flow cytometry [31].

### Flow cytometry

Peripheral blood from β-YAC and SCD mice was fixed in 4% paraformaldehyde, permeabilized in ice-cold acetone/methanol (4:1) and stained with fluorescein isothiocyanate (FITC) anti-HbF antibody (Life Technologies Corporation, Chicago IL; catalog #A80-136F) and FITC-isotype IgG antibody (Abcam, Waltham, MA; catalog #AB-37406). Flow cytometry was conducted on the FACSCanto machine (BD Biosciences, San Jose, CA) using gating parameters previously published by our group [28]. Briefly, the forward scatter and side scatter were set to gate erythroid cells, then during FLOWJO analysis, ‘log F-cell’‘ histograms (X-axis) generated to measure the percentage of HbF positive cells (F-cells) from total erythroid cells (Y-axis) after gating on the negative isotype control cells. The mean fluorescence intensity (MFI) to quantify the level of HbF protein per cell was generated by FLOWJO software.

### Reverse transcriptase-quantitative PCR (RT-qPCR)

The RT-qPCR conditions and specific primers used to measure globin gene mRNA were previously published by our group [30]. Total RNA was extracted from whole blood using Trizol Reagent (Life Technologies Corporation, Chicago IL; catalog #15596026), following the manufacturer’s protocol; then cDNA was synthesized using the ImProm-II™ Reverse Transcription System (Promega, Madison, WI; catalog #A3800) with oligo (dT)15 primers. RT-qPCR was performed in an SSO AdvancedTM Universal SYBR Green Super Mix (Bio-Rad, Hercules CA; catalog #172–5272) and CFX Connect Real-Time System (Bio-Rad) with gene-specific primers. All mRNA expression levels were normalized to glyceraldehyde-3-phosphate dehydrogenase (GAPDH) mRNA and calculated using standard curves.

### Spleen harvest

At the end of the 4-week treatment period, approximately 700 μL of blood was collected from each mouse into K3-EDTA tubes (Becton Dickinson, Franklin Lakes, NJ; catalog #365974) by cardiac puncture for plasma isolation after euthanasia using carbon dioxide gas. Subsequently, spleens were harvested for tissue stains, immunohistology, and protein isolation. A part of the spleen was fixed with buffered formaldehyde, embedded in paraffin, and sectioned into ~5mm thick slices for staining with hematoxylin and eosin for histology of spleen structures using a Keyence microscope (Keyence Itasca, IL). The immuno-histology staining was performed on frozen spleen sections using sheep polyclonal antibody against HbF conjugated with FITC (Life Technologies Corporation; catalog #A80-136F) and counterstained with DAPI (4′,6-diamidino-2-phenylindole) to identify the cell nucleus. The spleen tissue was analyzed using the EVOS cell imaging system (ThermoFisher, Waltham, MA) under x40 magnifications. The FITC-positive cells were counted for 15 high-power fields per section, and the mean average of the fields was used for statistical analysis.

For protein isolation, spleens tissue was dissociated into a single-cell suspension using the Spleen Dissociation Kit (Miltenyi Biotec, Auburn, CA; catalog #130-095-926), according to the manufacturer’s protocol, and a gentleman’s Octo Desiccator (Miltenyi Biotec, Auburn, CA). The total splenic cells were processed for histone acetylation levels by Western blot as previously published by our group [28]. In addition, ter-119^+^ cells were isolated from the total spleen cells with anti-ter119 microbeads (Miltenyi Biotec; catalog #130-120-828) using 10 μL/10^7^ spleen cells per the manufacturer’s instructions and RNA isolated for RT-qPCR analysis.

### Western blot

Acidic histone extraction was performed according to standard protocols described previously by our group [28,30]. The protein concentration was determined using the Bradford assay (Bio-Rad; catalog# 500–0205) at 595nm. Equal amounts (5 µg) of histone protein were loaded on an SDS–PAGE Mini-PROTEAN TGX 12% Gel (Bio-Rad; catalog #456–1043) and transferred onto a 0.2 μm PVDF membrane (Bio-Rad; catalog # 70–4157). After blocking with 5% non-fat dried milk, the membranes were incubated overnight at 4°C with primary antibody against total anti-histone H3 (Cell Signaling, Danvers, MA; catalog #4499S), anti-acetyl-histone H3 (MilliporeSigma St. Louis, MO; catalog #06–599), total anti-histone H4 (MilliporeSigma; catalog #07–108), and anti-acetyl-histone H4 (MilliporeSigma; catalog #06–598). Western blot quantitative data were collected by densitometry analysis using Image J software.

### Cytokine assay

Plasma was isolated from peripheral blood collected by cardiac puncture at the end of the treatment period to evaluate the cytokine and chemokine profiles in SCD mice. We used the MILLIPLEX MAP Mouse Cytokine/Chemokine Magnetic Bead Panel-Immunology Multiplex Assay (MilliporeSigma; catalog #MCYTOMAG-70K), which makes use of magnetic bead technology to quantify analytes, as described previously by our group [32].

### Micronuclei assay

After the whole blood was collected by cardiac puncture, the blood was centrifuged, plasma collected, and cell pellets washed in phosphate-buffered saline for micronuclei (MN) staining with CD71-FITC antibody (BD Biosciences, Milpitas, CA; catalog #553266) and 0.1mg RNase. DNA was stained with propidium iodide/phycoerythrin for simultaneous measurement of cell viability, followed by FACSCAN flow cytometry analysis. Propidium iodide/phycoerythrin supplied in the MN analysis kit (Litron Laboratories, Rochester, NY; catalog #PLUS-M) was used as a positive control for CD71-FITC; the number of MN positive cells were counted in cell positive for CD71 and MN as previously published [33].

### Statistical analysis

Descriptive statistics were generated with mean, standard deviation (SD) and standard error of the mean (SEM) for continuous variables and proportion for categorical variables. For all treatments, one-sample paired Student’s *t-tests* were performed to determine differences in complete blood counts, reticulocytes, F-cells, and MFI at week 0 compared to weeks 2 and 4 within groups (n = 8–12). A pairwise comparison analysis of variance (ANOVA) was performed to compare the effect across treatment groups. A level of *p* < 0.05 was used for statistical significance; all statistical analysis was performed in R 4.1.

## Results

### Development of drug formulations

The development of CT-101 for clinical use requires the design of a drug formulation that is effective by oral administration, however all studies performed in this report utilized IP administration to confirm HbF induction *in vivo*. Formulations using ethanol and n-methyl pyrrolidone were combined with different detergents (Tween-80, Solutol HS-15, Cavasol W7 HP), polyethylene glycol, and propylene glycol to screen for ideal candidates. Our β-YAC mice screening studies were performed with FORM-1, using ethanol (EtOH) as the principal solvent which was demonstrated to be stable at a concentration of 1.5 mg/ml CT-101 for up to 4 hours at room temperature.

In preparation for studies in the Townes SCD mouse model, we evaluated the tolerance to FORM-1 and demonstrated that the high EtOH content formulation was unsuitable as a vehicle. A second phase of formulation development used a lower EtOH content while keeping CT-101 solubility high (1.5–2.0 mg/mL) and generated three new candidate formulations (FORM-2, FORM-3, and FORM-4). All candidates were stable at room temperature and free from precipitation for up to 24 hours at CT-101 concentrations of 1.5–2.0 mg/mL and were subsequently shown to be well tolerated by Townes SCD mice.

**Fig 1:**
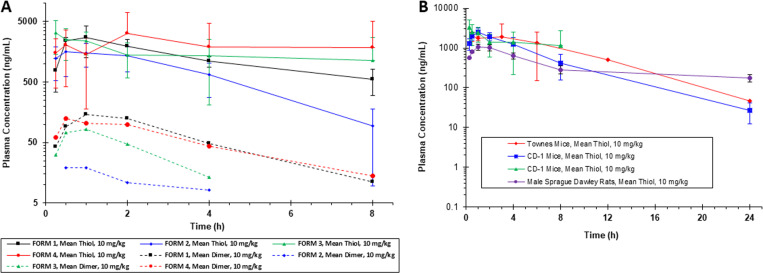
Development of drug formulation. To develop an optimal IP formulation, we screened drug candidates in pharmacokinetic (PK) studies to evaluate relative bioavailability. Four IP candidates were screened (Form-1, -2, -3, and -4) using CD-1 mice (n = 4); blood samples were collected over 8 hours and plasma prepared. **A)** An LC-MS method was used to determine CT-101S and CT-101 dimer levels and results were plotted as shown. **B)** PK profiles for CT-101S using the optimal IP formulation (FORM-3) in CD-1 mice at 8 hours (n = 4) and 24 hours (n = 4). PK profiles were compared to FORM-3 administered to Townes mice (n = 3) and male Sprague Dawley rats (n = 3). Results for total CT-101 (measured as CT101S) in the three groups were plotted.

The formulations were evaluated in an 8-hour bioavailability study in male CD-1 mice compared to FORM-1 as a single IP dose of CT-101 (10 mg/kg) for each group (n = 4 per group). Plasma samples collected at 15 min, 30 min, and 1, 2, 4, and 8 hours were evaluated for CT-101 analogue concentrations by LC-MS (Inotiv). Total CT-101 content determined as CT-101S, and CT-101 dimer were quantified and plasma decay kinetics plotted for the 8-hour study (Fig 1A). The raw data and variation within each group is summarized for CT-101S (S1 Table in S1 File) and CT-101 dimer (S2 Table in S1 File) and the significance is discussed further in Supporting Information Results.

Pharmacokinetics parameters were assessed and summarized for the 4 formulations evaluated in Supporting Information S3 Table in S1 File. The prodrug CT-101 was not quantified as it is undetectable 15–30 min after IP injection. CT-101 levels in plasma are primarily sensitive to reduction (measured as CT-101S), representing the analogue di-sulfide bound to plasma proteins. The most promising formulations were FORM-3 and FORM-4, with similar decay kinetics (Fig 1A), AUC_last_, and half-life (S3 Table in S1 File) in plasma and were comparable to FORM-1. We used FORM-3 for the Townes SCD mice studies since it was easier to prepare and administer than FORM-4.

### CT-101 bioavailability in SCD mice

The bioavailability of the prodrug CT-101 was evaluated in Townes SCD mice after a single IP injection at 10 mg/kg in FORM-3. Plasma samples were prepared at 15 and 30 min, and 1, 2, 4, 8, and 24 hours, stored frozen and shipped to Inotiv to quantify CT-101 analogues by LC-MS. Raw data for the 24-hour decay kinetics of CT-101S in plasma (reduced sample) in Townes SCD and CD-1 mice is summarized (S4 Table in S1 File) and shown in Fig 1B. CT-101 dimer data is not included since significant levels could not be detected for the 24-hour studies presented.

The two additional data sets included in Fig 1B were part of an oral formulation program where a FORM-3 control arm was included and a second study using male Sprague Dawley rats for a 24-hour bioavailability study. Both studies dosed CT-101 in FORM-3 by intraperitoneal injection at 10 mg/kg. Pharmacokinetics parameters for the 4 data sets are summarized in the Supporting Information S5 Table in S1 File. The calculated AUC for CT-101S in SCD mice (n = 3) was 18,8000 (hr x ng/mL) compared to an average of ~ 13,000 for CD-1 mice (n = 4). The half-life of CT-101 in all mouse groups was ~ 5 hours, and no significant difference in plasma levels of the drug was observed in SCD mice. The results demonstrate a similar uptake and PK profile for CT-101 in all groups evaluated. Pharmacokinetic parameters for mice and rats compared well given xenometric scaling for both species [34]. Additionally, the rat study data supports the data generated in mice suggesting there may be a longer half-life for CT-101 in higher animals.

### CT-101 activates γ-globin gene transcription and HbF production in β-YAC mice

**Fig 2:**
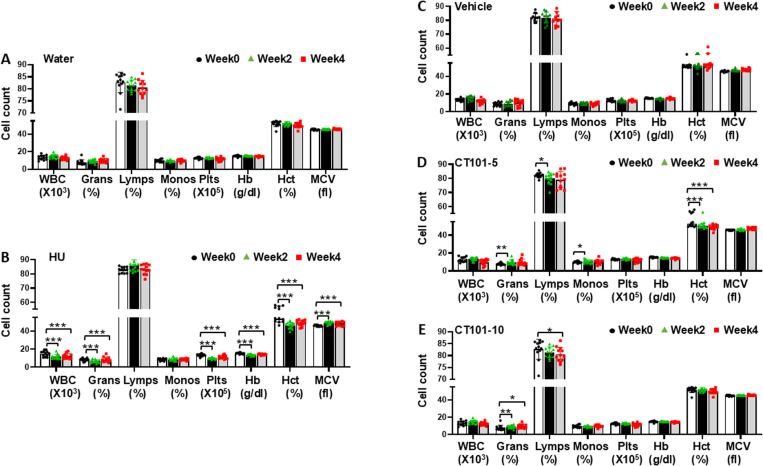
The effects of CT-101 on complete blood cell counts and differentials forβ-YAC mice. To determine the optimal dose of CT-101 free of cellular toxicity, we treated β-YAC mice with CT-101 (5 and 10 mg/kg) by intraperitoneal (IP) injections three days a week for four weeks. Peripheral blood samples were collected for automated complete blood counts and differentials using a Micros 60 machine on week 0 (baseline), week 2, and week 4. For all graphs shown in Fig 2, n = 12 mice in each treatment group. Analysis included **A)** vehicle (water), **B)** hydroxyurea, **C)** vehicle (FORM-1), **D)** CT101 5 mg/kg, and **E)** CT-101 10 mg/kg. Abbreviations: WBC, white blood cell count; Grans, granulocytes, Lymps, lymphocytes; Monos, monocytes; Plts, platelets; RBC, red blood cells; Hb, hemoglobin; MCV, mean corpuscular volume; MCH, mean corpuscular hemoglobin; RDW, red cell distribution width. Data are shown as the mean ± standard error of the mean (SEM). Symbols: * = p < 0.05, ** = p < 0.01, *** = p < 0.001.

Initial studies determined the ability of CT-101 to activate γ-globin gene transcription and induce HbF expression *in vivo,* using β-YAC transgenic mice. Study 1 involved once daily dosing of CT-101 at 5 and 10 mg/kg IP injections, three days a week (M, W, F) and HU (100 mg/kg), five days a week (M-F) for four weeks. Mouse weights were monitored weekly, along with laboratory evaluations at weeks 0, 2, and 4, to assess the effects of CT-101 on peripheral blood cell counts, globin gene transcription, and the percentage of red blood cells (RBCs) positive for HbF (F-cells). As has been observed in previous studies with β-YAC mice [35] and individuals with SCD [5,8], HU significantly decreased total white cell counts (24%), platelets (30%), and hemoglobin and hematocrit by 12% while increasing the mean corpuscular volume (p < 0.001) of RBCs (Fig 2A and 2B); we also observed a concomitant increase in reticulocyte counts produced by HU (S1 Figure in S1 File). By contrast, both CT-101 doses mediated a mild decrease in lymphocytes and hematocrit however none of the other blood cell parameters showed clinically significant changes during treatment (Fig 2C–2E).

**Fig 3:**
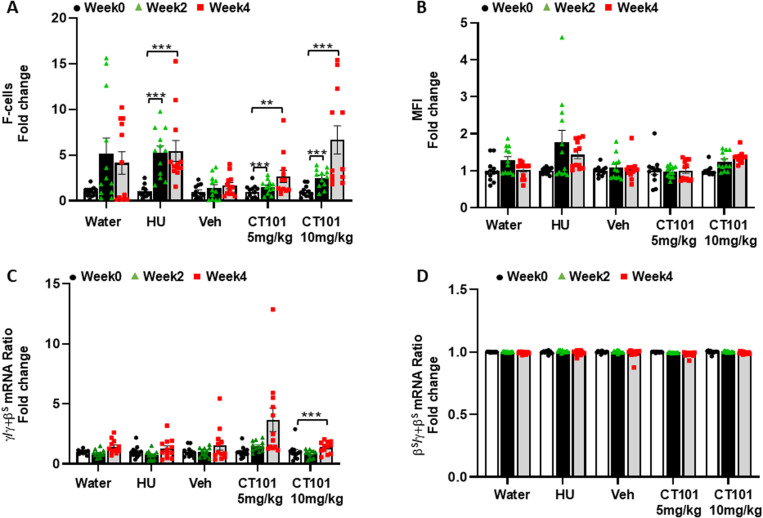
CT-101 increasedγ-globin gene transcription and F-cell levels in β-YAC mice. To determine the optimal dose of CT-101, which mediates HbF induction, flow cytometry, and RT-qPCR analyses were completed on peripheral blood. β-YAC mice were treated with CT-101 5 and 10 mg/kg by IP injections once daily, three days a week for four weeks. For all graphs, n = 12 mice per treatment group. Peripheral blood samples were collected for **A)** flow cytometry to determine the percentage of F-cells at weeks 0, 2, and 4. Analysis was done for the treatment groups described in the figure. **B)** Flow cytometry was completed to determine the HbF level per cell by MFI. **C)** RT-qPCR was performed to determine the level of γ-globin mRNA as the γ/γ+β globin mRNA ratio and **D)** β-globin gene transcription calculated as β/γ+β globin mRNA. Symbols: * = p < 0.05, ** = p<0.01, *** = p < 0.001.

Next, we performed flow cytometry to measure the percentage of F-cells and the level of HbF protein level per cell using MFI analysis of peripheral blood. By week 4, we observed a dose-dependent significant 2.7-fold (p < 0.01) and 6.7-fold (p < 0.001) increase in F-cells by CT-101 at 5 and 10 mg/kg, respectively, compared to a 5.4-fold (p < 0.001) increase in F-cells produced by HU (Fig 3A). However, there was no significant difference across treatment groups by ANOVA. By contrast, no significant increases in MFI were observed at both CT-101 doses (Fig 3B). At the mRNA level, CT-101 5 mg/kg activated γ-globin gene transcription (measured by the ratio of γ-globin mRNA divided by total globin mRNA levels) 1.5-fold and 3.7-fold for weeks 2 and 4 respectively, but due to large variations in individual responses these values were not statistically significant (Fig 3C); at week 4 the CT-101 10 mg/kg dose produce a significant 1.5 fold (p < 0.001) increase in γ-globin mRNA levels. By contrast there were no significant changes β-globin gene mRNA levels (Fig 3D). These results suggest that CT-101 preferentially activates γ-globin gene transcription as a mechanism of HbF induction.

**Fig 4:**
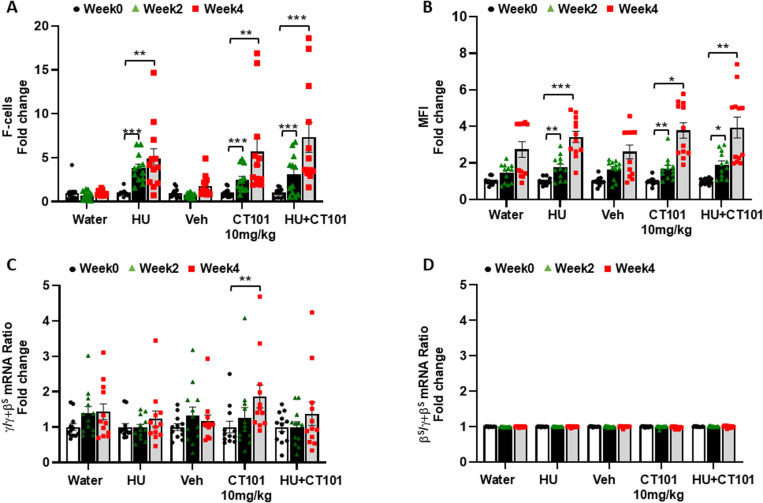
Combined HU and CT-101 treatment inβ-YAC mice. To determine the effects of combination treatment, in Study 2 we evaluated CT-101 10 mg/kg combined with HU 100 mg/kg, for four weeks. For all graphs n = 12 mice per treatment group. Peripheral blood samples were collected for **A)** flow cytometry to determine the percentage of F-cells in weeks 0, 2, and 4. Treatment groups are labeled. **B)** Flow cytometry determines the MFI levels. **C)** RT-qPCR was performed to determine the γ/γ+β globin mRNA ratio and **D)** β-globin gene transcription calculated as β/γ+β mRNA ratio. Symbols: * = p < 0.05, ** = p<0.01, *** = p < 0.001.

Since HU and CT-101 work by different molecular mechanisms and we observed an additive effect on HbF expression for our *in vitro* sickle erythroid progenitor studies [25], Study 2 was conducted to determine if combination treatment would produce additive effects on HbF synthesis *in vivo*. In subsequent studies, β-YAC mice were treated with CT-101 at 10 mg/kg three times a week or combined with HU (100mg/kg) by IP injections five days a week for four weeks. Analysis in weeks 0, 2, and 4 showed limited effects on peripheral blood counts mediated by CT-101 alone which produced no adverse effects (S2 Figure in S1 File). At the same time, we observed a decrease in total white blood counts and platelets produced by HU (S2 Figure in S1 File). Interestingly, when the two agents were combined, the significant repressive effects of HU on white cells and platelets were inhibited by adding CT-101 (S2E Figure in S1 File). By week 2, CT-101 10 mg/kg mediated a 2.4-fold (p < 0.001) increase in F-cells and 1.8-fold (p < 0.01) increase in MFI levels (Fig 4A and 4B). Moreover, in week 4, HbF induction continued to rise to 5.6-fold (p < 0.01) and 1.9-fold (p < 0.001) for F-cells and MFI, respectively. This is compared to a 7.3-fold (p < 0.001) and 3.9-fold (p < 0.01) increase in F-cells and MFI respectively for combined HU/CT-101 treatment at week 4 (Fig 4A and 4B) supporting an additive effect. As shown in Fig 4C, at the molecular levels CT-101 activated γ-globin mRNA synthesis significantly by 1.9-fold (p < 0.01) with no significant change β-globin mRNA levels for any treatment group (Fig 4D). Studies 1 and 2 in β-YAC mice provided *in vivo* evidence of the ability of CT-101 10 mg/kg to consistently activate γ-globin gene transcription and HbF protein synthesis and produce an additive effect with HU under non-oxidative stress conditions.

### CT-101 activates γ-globin gene transcription in SCD mice

The Townes SCD mouse model reliably recapitulates the clinical symptoms observed in patients with SCD, including chronic hemolysis, oxidative stress, organ damage, and splenomegaly, among others [36]. Since SCD mice are fragile and may not tolerate the same drug dose as healthier β-YAC mice, we performed drug toxicity studies. As described above, different vehicle compositions were evaluated; dosing CT-101 in FORM-3 for a week, produced limited toxicity in SCD mice allowing us to move forward with our studies under oxidative stress conditions. Using a similar experimental design of once daily IP dosing, SCD mice were treated with CT-101 10 mg/kg (M, W, F), HU (100 mg/kg) five times a week, or combined CT-101/HU treatment, for four weeks along with water and vehicle (FORM-3) control groups.

CT-101 treatment was well tolerated in SCD mice with good weight gain and behavior throughout the study period (S3A Figure in S1 File). We tracked survival for two independent studies (n = 8–12) and observed unexpected mortality during the first replicate of 67% for CT-101 alone and 16% for HU/CT-101 combined. However, for replicate two, we observed 100% survival for the CT-101 alone treatment group (S3B and S3C Figures in S1 File), suggesting there may have been technical difficulties with the first replicate. When both replicates were combined, 70% and 83% survival was observed for CT-101 and vehicle groups, compared to 83% and 100% survival for HU and water groups, respectively.

**Fig 5:**
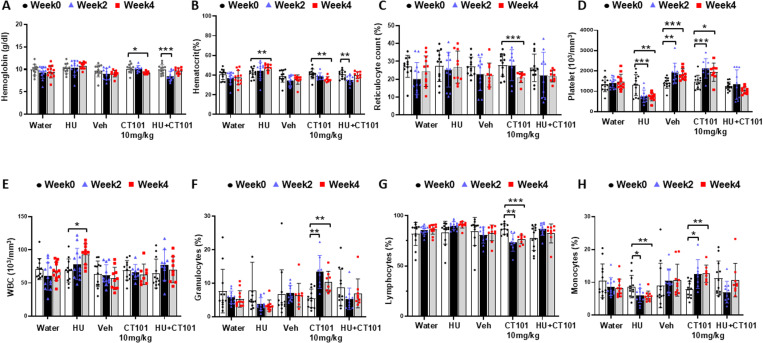
CT-101 produces minimal effects on peripheral blood counts in SCD mice. To confirm the ability of CT-101 to induce HbF under oxidative stress conditions, we completed Study 3 using the Townes SCD mouse model, requiring a new drug formulation solvent FORM-3. SCD mice were treated with water, hydroxyurea (HU), FORM-3 (Veh), CT-101 10 mg/kg (CT-101), and combined HU 100 mg/kg and CT-101 10 mg/kg (HU/CT-101) by IP injections for four weeks (n = 12 mice per treatment group). Peripheral blood was collected for automated complete blood counts and differentials at weeks 0, 2, and 4. Blood was also stained with acridine orange to measure changes in reticulocyte counts by flow cytometry analysis. Deaths occurred during the study, so by week 4 we evaluated for water (n = 12), HU (n = 10), Veh (n = 10), CT-101 (n = 8) and combination therapy (n = 9). Shown are levels of: **A)** hemoglobin, **B)** hematocrit, **C)** reticulocyte counts, **D)** platelet counts, **E)** white blood cells, **F)** granulocytes, **G)** lymphocytes, and **H)** monocytes. Symbols: * = p < 0.05, ** = p<0.01, *** = p < 0.001.

To determine the overall effects of CT-101 on hematopoiesis in SCD mice, we monitored complete blood counts with differentials, and reticulocyte counts at 2-week intervals. CT-101 alone or combined with HU produced a decrease in hemoglobin and hematocrit in SCD mice which had no effect on the activity of mice during the treatment period (Fig 5A and 5B). Interestingly, in week 4 CT-101 produced a significant 24% decrease (p < 0.001) in reticulocyte percentages (Fig 5C); as previously published by our group [37], HU significantly decreased platelet counts by 50% (p < 0.01) throughout the study (Fig 5D) but there were no signs of increased bleeding risk in SCD mice. This contrasted with the significant increase in platelet counts for vehicle and CT-101 treatment suggesting a placebo effect.

We next reviewed the effects of drug treatment on the total white blood cell and differential counts. Of note, CT-101 alone or combined with HU did not produce changes in the total white cell counts (Fig 5E). By contrast, CT-101 increased granulocytes significantly (p < 0.01) in weeks 2 and 4 while decreasing lymphocytes by 15% (Fig. F-H). Lastly, we observed a compensatory 1.6-fold (p < 0.01) increase in monocytes in response to decrease lymphocyte production mediated by CT-101 that was reversed by combination treatment (Fig 5G and 5H).

**Fig 6:**
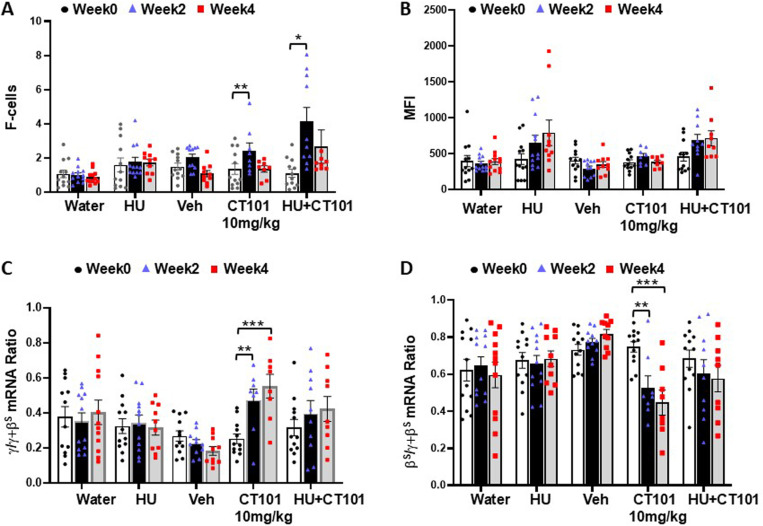
CT-101 enhancesγ-globin gene transcription in SCD mice. To determine the ability of CT-101 to induce HbF in SCD mice, flow cytometry and RT-qPCR analyses were completed in week 0 for n = 12 mice per treatment group. Deaths were observed during the study, thus by week 4 we evaluated water (n = 12), hydroxyurea (HU; n = 10), FORM-3 (Veh; n = 10), CT-101 10 mg/kg (CT-101; n = 8), and combined HU/CT-101 (n = 9). Peripheral blood samples **A)** determined the percentage of F-cells and **B)** the HbF level per cell by MFI. **C)** RT-qPCR determined the change in γ-globin gene transcription calculated as γ/γ+β^S^ mRNA ratio and **D)** β^S^-globin gene transcription calculated as β^S^/γ+β^S^ mRNA ratio. Symbols: * = p < 0.05, ** = p<0.01, *** = p < 0.001.

To assess the ability of CT-101 to induce HbF and activate γ-globin gene transcription in SCD mice, flow cytometry and RT-qPCR analyses were performed. CT-101 (10 mg/kg) produced a significant 2.1-fold (p < 0.01) increase in F-cells and an additive 3.7-fold (p < 0.05) effect when combined with HU by week 2 (Fig 6A); by contrast, the MFI remained unchanged for all treatment groups (Fig 6B). Lastly, mRNA levels demonstrated preferential activation of γ-globin gene transcription a maximal 1.9-fold (p < 0.001) with a simultaneous 39% (p < 0.001) decrease in β^S^-globin mRNA levels by CT-101 in week 4 of treatment (Fig 6C and 6D).

### CT-101 mediates erythrocyte lineage maturation and decreased hemorrhagic infarcts in spleen tissue of SCD mice

**Fig 7:**
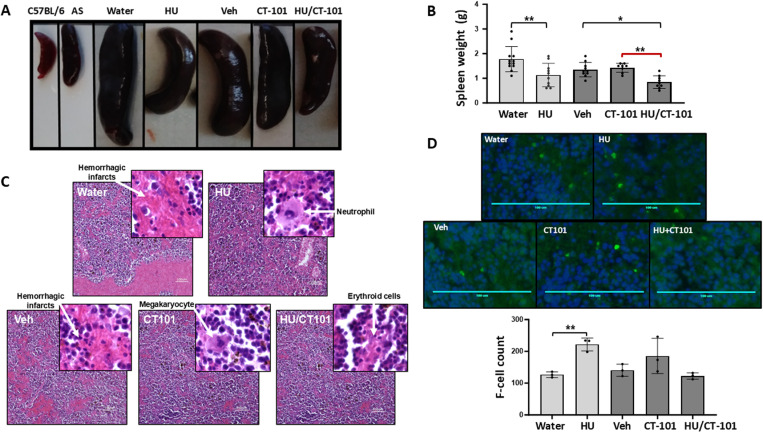
Spleen tissue analysis in SCD mice. At the end of treatment, SCD mice were sacrificed for spleen harvest. **A)** The gross images of the spleens at four weeks in the different treatment groups are shown. Splenomegaly is evident in mice treated with water or FORM-3 controls, with a reduction in splenomegaly of varying degrees in the treatment groups including hydroxyurea (HU), CT101 10 mg/kg (CT-101), and combined HU 100 mg/kg and CT-101 10 mg/kg (HU/CT-101); untreated C57BL/6 and sickle cell trait (AS) mice spleens are shown for comparison. **B)** The graph summarizes the different treatment group spleen weights for water (n = 12), HU (n = 10), Veh (n = 10), CT-101 (n = 8), and HU/CT101 (n = 9). **C)** Histology sections were prepared for hematoxylin and eosin staining. Representative slides at 10x magnification are shown for the different treatment groups; scale bar equals 100 micrometers. The image insets represent 80x magnification. We observed an increase in lymphoid elements, maturing extramedullary hematopoiesis (megakaryocytes and erythroid cells), and decreased hemorrhagic infarcts for splenic tissue from HU, CT-101, and HU/CT-101 treated groups. By contrast, the water and vehicle control groups showed hemorrhagic infarct and ischemia. **D)** Representative slides of anti-FITC-HbF stained spleen tissue at 40x magnification for the different treatment groups are shown (n = 3 per group); scale bar equals 100 micrometers. Symbols: * = p < 0.05, ** = p<0.01, *** = p < 0.001.

Our last set of experiments in SCD mice involved harvesting spleen tissue and blood at the end of treatment to examine tissue structure and perform plasma cytokine, micronuclei count, and nuclear histone acetylation levels. Fig 7A and 7B show that overall spleen size significantly decreased by 37% for HU (p < 0.01); moreover, HU/CT-101 treatment showed a significant 20% (p < 0.05) decrease in spleen size compared to vehicle. Additional ANOVA data demonstrated combined HU/CT-101 treatment significantly decreased the spleen size about 42% (p < 0.01; red bracket) compared to CT-101 alone. Subsequent spleen tissue staining with hematoxylin and eosin showed a decrease in hemorrhagic infarcts and maturing extramedullary hematopoiesis mediated by HU, CT-101, and combined treatments (Fig 7C, white arrows). To further assess HbF expression, spleen tissue was stained with anti-fluorescein isothiocyanate (FITC)-HbF antibody, demonstrating HU produce a significant 1.8-fold (p < 0.01) increase in HbF-positive erythroid cell as shown in Fig 7D; the increase in HbF positive cells produced by CT-101 was not significant.

We finally quantified plasma cytokine levels using magnetic bead-based multiplex studies where we observed no significant change in interleukin 6 (IL-6), IL10 or the neutrophil-attractant KC chemokine levels (Fig. S4A-C in S1 File). We quantified micronuclei levels, which showed a significant 1.8-fold (p < 0.05), and 1.5-fold (p < 0.05) increased production in HU, or combined HU/CT-101 treatment respectively, compared to no significant changes mediated by CT-101 alone (S4D Figure in S1 File). To determine the effects of CT-101 on nuclear histone acetylation levels, protein isolated from spleen tissue was processed by Western blot analysis that showed no significant changes in total or acetylated histone H3 and H4 levels (S5 Figure in S1 File).

## Discussion

Sickle cell disease remains a significant health burden in the US and is one of the most prevalent inherited blood disorders worldwide. However, HU is the only HbF-inducing agent currently approved by the US FDA and recommended as the standard of care for individuals with SCD. Subsequent, approval of non-curative drugs including L-glutamine [12], crizanlizumab [13], and voxelotor [14], provided SCD patients with alternatives to manage acute vaso-occlusive pain episodes and anemia. However, these agents have experienced slow clinical uptake and recently, voxelotor was abruptly withdrawn from the market due to safety concerns. To date, the only curative approach for SCD is hematopoietic stem cell transplantation with matched sibling or unrelated donors. However, this option is limited by the availability of suitable donors [38]. Although there is a demonstrated greater than 80% event-free survival reported [39], the complications of graft versus host disease, infection, infertility, and death are considerable. Hence, efforts to address these complications are under investigation [40].

With the introduction of effective lentivirus-based gene therapy vectors and gene editing using CRISPR technology and other novel approaches, there have been efforts to expand curative options for individuals with SCD. After decades of research, the US FDA approved two gene therapy drugs for SCD in December 2023. The first is Exa-cel, using CRISPR technology to disrupt an erythroid-specific enhancer in *BCL11A*, a significant repressor of γ-globin gene transcription [41,42]. Clinical trials showed Exa-cel produced ~50% homogenous HbF activation and significant resolution of clinical vaso-occlusive pain episodes [42]. The second approved drug is Lovo-cel [43], which carries an anti-sickling hemoglobin HbA^T87Q^ in a lentivirus vector. This agent was initially evaluated in transfusion-dependent β-thalassemia patients, demonstrating 91% of patients achieving transfusion independence [44]. Subsequent clinical trials in SCD patients demonstrated almost complete resolution of vaso-occlusive pain episodes [43]. Questions remain unanswered about the safety of CRISPR technology related to off-target effects and random genomic integration of lentivirus vectors. With limited clinical follow-up, the question of whether gene therapy will produce a lifelong cure remains unanswered. Additionally, initial estimates of the treatment costs are between $2.2 and $3 million, limiting access for SCD patients in the US and low-income countries. There remains a need to develop safe and effective low-cost oral drugs to treat SCD.

To develop other effective HbF inducers, we expanded our initial *in vitro* findings [25] by examining the ability of the small molecule Class I HDAC inhibitor, CT-101, to induce HbF *in vivo*. Our first studies using β-YAC mice established the optimal dose and ability of CT-101 to increase F-cells, MFI, and γ-globin gene transcription. Subsequent studies in Townes SCD mice showed minimal effects of CT-101 on peripheral blood cell counts while mediating increases in γ-globin gene transcription and F-cell levels and β^S^-globin gene silencing. We next asked whether combined treatment with CT-101 and HU would produce an additive effect on HbF induction since these agents work by different molecular mechanisms. Although combined CT-101/HU treatment produce an additive effect on F-cells in β-YAC mice, the same was not observed in SCD mice. Interestingly, treatment with HU alone or combined CT-101/HU produced a significant decrease in spleen size and stimulated maturing extramedullary hematopoiesis, suggesting potential clinical benefits of adding CT-101 to HU treatment. Finally, plasma cytokine and histone acetylation studies were not significantly changed by CT-101 which may be due to small sample size and/or analysis of whole spleen tissue. For CT-101 to advance to clinic studies an oral formulation of the drug will need to be developed.

Historically, drugs that alter epigenetic marks, such as HDAC and DNA methyltransferase inhibitors, have been investigated as options to increase HbF levels, which effectively blocks hemoglobin S polymerization and ameliorate clinical symptoms of SCD [45]. Multiple transcriptional and epigenetic regulators of γ-globin gene transcription, including MBD-2, BCL11A, KLF1, and LRF mediate HbF silencing during hemoglobin switching [29,46–55]. Initial family genetic studies [56] followed by a genome-wide association study [57] discovered BCL11A as a major repressor of γ-globin transcription, accounting for ~ 30% of HbF level variations in adults with SCD. Later studies demonstrated that BCL11A mediates the interactions of the Class 1 HDAC1 and HDAC2 in the NuRD complex [47,58,24]. Our data in sickle erythroid progenitors [25] demonstrated that CT-101 produced minimal cell toxicity, like previous studies using HDAC1 and HDAC2 inhibitors [58], which have no effects on cell proliferation or cell cycle phase. We observed γ-globin gene activation and β^S^-globin gene silencing, which supports findings previously published in erythroid progenitors treated with suberoylanilide hydroxamic acid [59], pan-cellular HDAC inhibitors [60] and the HDAC1 and HDAC3 inhibitor, MS-275 [46].

A significant unmet need remains to develop additional low-cost oral small molecules to treat SCD. to expand the repertoire of agents that induce HbF, historically, butyrate [61] and decitabine [5] produced robust HbF induction by intravenous route, but efficacy was lost when administered orally. Previous clinical trials [21] show promise when decitabine is combined with the cytosine deaminase inhibitor, tetrahydrouridine, but required two medications to be taken sequentially, producing challenges with long-term treatment adherence. More recently, pharma, in collaboration with academic investigators, is developing a combination capsule to solve this dilemma. Additional oral HbF-inducing agents in clinical trials include the DNA methyl transferase 1 depletor AB1 [62], benezeraside (PB-04) [63], FTX-6058 (PRC2 inhibitor) [64], and the pan-cellular HDAC inhibitor panobinostat [65].

With the declaration of SCD as a world health problem by the World Health Organization in 2010, global treatment efforts to decrease morbidity and mortality have been expanded in Sub-Saharan Africa, Brazil, and India, where most people with SCD live. Realizing Effectiveness Across Continents with Hydroxyurea (REACH) is an open-label prospective trial of HU for children in sub-Saharan Africa countries (Angola, Democratic Republic of Congo, Kenya, and Uganda). An 8-year follow-up period was recently evaluated where HU was safe, well-tolerated, and effective for children [66]. Furthermore, HU escalation to maximal tolerated dose improved laboratory and clinical responses without increasing toxicity. The 30–80% mortality rate of SCD in children under five years of age in sub-Saharan Africa justifies the development of additional low-cost oral effective small molecules that require minimal monitoring to decrease the morbidity and mortality of SCD worldwide.

### Limitations

Overall, the Class 1 HDAC inhibitor CT-101 was well tolerated and induced HbF expression under low oxidative stress conditions in β-YAC mice and high oxidative stress in SCD mice suggesting its promise for future development in humans. Our studies utilized an intraperitoneal formulation to administer CT-101 in SCD mice where toxicity of the FORM1 vehicle was observed requiring development of a different vehicle. While acceptable as a formulation for research, commercialization of CT-101 for use in adults with SCD will require the design of an effective and safe oral formulation. When CT-101 was administered to animals, the rapid formation of a free thiol metabolite CT-101S occurred which binds to plasma proteins via di-sulfide bonds. Studies examining CT-101S binding to plasma proteins after administration of CT-101 would directly determine the extent and level of these interactions and ensure the drug is safe to use in humans.

To establish the clinical efficacy of CT-101 we performed different studies that support γ-globin gene activation at the mRNA level and HbF protein induction in RBCs as demonstrated by RT-qPCR and flow cytometry, respectively. However, we did not observe HbF induction by MFI analysis suggesting other methods such as HPLC might be a better approach to quantify levels. Another limitation was our ability to show histone H3 and H4 acetylation after CT-101 treatment. Spleen tissue is a mixture of cells from all hematopoietic lineages therefore demonstrating an increase in histone acetylation may require purification of TER119^+^ erythroid progenitors for RT-qPCR analysis in future studies. The sample size for the cytokine analysis will need to be increased to evaluate the effects of CT-101 in the plasma and fully elucidate its ability to produce a desirable anti-inflammatory effect.

## Conclusion

The studies presented in this report demonstrate the utility of a novel small molecule inhibitor of Class I HDAC isoforms involved in γ-globin gene silencing via the NuRD complex. CT-101 was well tolerated and selectively increased γ-globin gene transcription and HbF synthesis in SCD mice without significant toxicity to peripheral blood cell counts. While curative approaches for SCD are available and challenges to developing an oral formulation for CT-101 exist, we believe there remains a need to investigate additional drugs that can flip the γ-globin to β-globin gene switch that are well tolerated, efficacious and affordable for global use.

## Supporting information

S1 File S1-S5 Tables and S1-S5 Figures (PDF)

S1 Raw images Original Western Blot Gels (PDF)
