# Supplementary material for: The histone deacetylase inhibitor CT-101 flips the switch to fetal hemoglobin expression in sickle cell disease mice
Source: PLoS One. 2025 May 13;20(5):e0323550. doi: 10.1371/journal.pone.0323550 (PMC12074596; doi:10.1371/journal.pone.0323550)
Supplement: S1 File — (PDF) [file pone.0323550.s001.pdf]

SUPPORTING INFORMATION

The Histone Deacetylase Inhibitor CT-101 Flips the Switch to Fetal Hemoglobin Expression in Sickle Cell Disease Mice

Mayuko Takezaki*[1], Biaoru Li*[1], Hongyan Xu[2], Nikhil Patel[3], Rudolf Lucas[4], Ryan Cerbone[5,6], Sivanagireddy Koti[5], Clifford L. Hendrick[5], Louis H. Junker**[5], Betty S. Pace**[1]

[1]Department of Pediatrics, Georgia Cancer Center, Augusta University, Augusta, GA 30912

[2]Department of Biostatistics, Data Science and Epidemiology, Augusta University, Augusta, GA 30912

[3]Department of Pathology and Laboratory Medicine, Medical College of Georgia at Augusta University, Augusta, GA 30912

[4]Vascular Biology Center, Department of Pharmacology and Toxicology, Division of Pulmonary and Critical Care Medicine, Medical College of Georgia at Augusta University, Augusta, GA 30912

[5]Cetya Therapeutics, Fort Collins, CO 80524

[6]Colorado State University, Department of Chemistry, Fort Collins, CO 80523

Supporting Materials and Methods

Bioavailability and pharmacokinetic studies in animals

Pharmacokinetic (PK) analysis included the determination of maximum concentration (C_{max}), time of maximum concentration (T_{max}), area under the curve (AUC_{last}), the final time point analyzed (T_{last}), and half-life ($T_{1/2}$) and values reported. The mean CT-101S and CT-101 dimer plasma concentrations and CV% were determined. PK parameters were presented as individual replicates and mean values. Each data point was presented to the number of decimal points shown.

Supporting Results

Development of drug formulations

Data supporting Fig. 1A and Fig. 1B was analyzed, and the average, standard deviation (SD), and relative SD (RSD) calculated for CT-101S (Table S1) and CT-101 dimer (Table S2). The lowest variability for CT-101S was FORM-1 (37%), followed by FORM-2 (59%), FORM-3 (73%), and FORM-4 (113%) (Table S1). The variability of the CT-101 dimer concentrations was higher due to lower concentrations of this analog, with RSD values ranging from 95% to 139% (Table S2). Study PK parameters were calculated, and C_{max} , T_{max}, AUC_{last}, T_{last}, and $T_{1/2}$ reported (Table S3).

Supporting Tables

S1 Table. Individual data points, average, standard deviation (SD) and relative standard deviation (RSD) supporting plasma CT101S (Figures 1A). *							
**Time (hr)**	**FORM-1 Plasma CT101S**				**Average**	**SD**	**RSD**
**0.25**	ISV	1,260	636	425	774	434	56%
**0.5**	2,320	2,230	2,020	2,860	2,358	358	15%
**1**	2,180	1,880	4,930	1,950	2,735	1,469	54%
**2**	1,730	2,180	2,560	1,290	1,940	550	28%
**4**	1,280	1,300	877	930	1,097	224	20%
**8**	709	794	230	474	552	254	46%
**Average RSD**							**37%**
	**FORM-2 Plasma CT-101S**				**Average**	**SD**	**RSD**
**0.25**	1,030	573	1,090	2,200	1,223	691	56%
**0.5**	829	700	2,090	2,670	1,572	964	61%
**1**	925	2,290	1,080	1,770	1,516	633	42%
**2**	621	1,990	1,650	1,090	1,338	605	45%
**4**	219	907	835	ISV	654	378	58%
**8**	37.4	217	67.6	50.4	93	84	90%
**Average RSD**							**59%**
	**FORM-3 Plasma CT-101S**				**Average**	**SD**	**RSD**
**0.25**	4,100	984	5,280	2,680	3,261	1,853	57%
**0.5**	1,580	2,270	4,430	1,640	2,480	1,337	54%
**1**	2,310	3,310	2,860	1,030	2,378	987	42%
**2**	1,030	2,590	1,250	748	1,405	817	58%
**4**	819	3,090	922	629	1,365	1,156	85%
**8**	706	3,500	254	75	1,134	1,600	141%
**Average RSD**							**73%**
	**FORM-4 Plasma CT-101S**				**Average**	**SD**	**RSD**
**0.25**	2,340	958	204	2,450	1,488	1,093	73%
**0.5**	2,410	1,410	248	4,050	2,030	1,611	79%
**1**	1,950	794	66	2,880	1,423	1,243	87%
**2**	2,720	8,630	BLQ	1,460	3,203	3,785	118%
**4**	766	6,000	12	775	1,888	2,764	146%
**8**	200	6,630	5	528	1,841	3,200	174%
**Average RSD**							**113%**

*All concentrations in **ng/mL**
Abbreviations: BLQ, below limit of quantitation; **ISV,** insufficient volume to perform assay; **ND,** not done

**S2 Table. Individual data points, average, standard deviation (SD) and relative standard deviation (RSD) supporting CT101 dimer (Figures 1A). ***						
**FORM-1 Plasma CT101 Dimer**				**Average**	**SD**	**RSD**
ISV	41.8	ISV	ISV	42	ND	ND
22.8	159	ISV	ISV	91	ND	ND
ISV	264	88.9	86.7	147	102	69%
ISV	333	BLQ	45	126	181	143%
BLQ	170	BLQ	22.2	48	82	171%
18.1	26.6	BLQ	BLQ	11	13	120%
**Average RSD**						**126%**
**FORM-2 Plasma CT101 Dimer**				**Average**	**SD**	**RSD**
BLQ	BLQ	ISV	BLQ	BLQ	ND	ND
12.5	24.1	10.2	29.4	19	9	48%
ISV	34.6	BLQ	22.8	19	18	92%
BLQ	43.6	BLQ	BLQ	11	22	200%
BLQ	24.6	BLQ	ISV	8	14	173%
BLQ	BLQ	BLQ	ISV	BLQ	ND	ND
**Average RSD**						**128%**
**FORM-3 Plasma CT101 Dimer**				**Average**	**SD**	**RSD**
25.5	26.1	35.9	38.0	31	6	21%
49.1	92.5	67.2	80.5	72	19	26%
38.0	156.0	88.4	47.1	82	54	65%
BLQ	161.0	25.5	BLQ	47	77	166%
BLQ	54.0	BLQ	BLQ	14	27	200%
BLQ	BLQ	BLQ	BLQ	BLQ	ND	ND
**Average RSD**						**95%**
**FORM-4 Plasma CT101 Dimer**				**Average**	**STD**	**RSD**
95.2	17.8	BLQ	132.0	61	63	102%
162.0	60.0	BLQ	281.0	126	123	98%
91.1	ISV	BLQ	217.0	103	109	106%
18.5	330.0	BLQ	48.3	99	155	156%
0.0	174.0	BLQ	BLQ	44	87	200%
/ISV	41.8	BLQ	BLQ	14	24	173%
**Average RSD**						**139%**

*All concentrations in **ng/mL**
Abbreviations: BLQ, below limit of quantitation; **ISV,** insufficient volume to perform assay; **ND,** not done

S3 Table. Summary of pharmacokinetics raw data for plasma CT-101 thiol and CT101 dimer levels measured by LC-MS.

FORM	CT-101 Analogue	Parameter	C_{max}	T_{max}	AUC_{last}	T_{last}	$T_{1/2}$	Number of Data Points
			(ng/mL)	(hr)	(hr*ng/mL)	(hr)	(hr)	
1	Thiol	Mean	3,090	0.6	10,500	8	3.7	n = 4
	Thiol	SD	1,260	0.3	1,480	0	1.3	
	Thiol	%RSD	41	40.0	14	0	35	
	Dimer	Mean	210	1.5	754	6	1.8	n = 2
2	Thiol	Mean	2,020	0.6	6,250	8	1.5	n = 4
	Thiol	SD	702	0.3	2,310	0	0.3	
	Thiol	%RSD	35	55.9	37	0	18	
	Dimer	Mean	44	2.0	125	4	2.4	n = 1
3	Thiol	Mean	3,890	2.2	12,000	8	5.3	n = 4
	Thiol	SD	1,090	3.9	8,110	0	5.5	
	Thiol	%RSD	28	177	68	0	104	
	Dimer	Mean	95	1.0	163	2	1.0	n = 4
	Dimer	SD	47	0.7	197	1.4	0.4	
	Dimer	%RSD	50	70.7	121	71	43	
4	Thiol	Mean	3,910	1.3	16,300	8	2.6	n = 4
	Thiol	SD	3,520	0.9	20,000	0	1.5	
	Thiol	%RSD	90	69	122	0	58	
	Dimer	Mean	258	1.0	576	4	1.0	n = 3
	Dimer	SD	86.4	0.9	581	3.5	0.9	
	Dimer	%RSD	33.5	86.6	101	87	85	

Abbreviations: C_{max}, maximum concentration; T_{max}, time of maximum concentration; AUC_{last}, area under the curve; T_{last}, final time point; $T_{1/2}$, half-life; **RSD,** relative standard deviation.

S4 Table. Individual data points, average, standard deviation (SD) and relative standard deviation (RSD) supporting CT101S in mice and rats (Figures 1C).*							
**Time (hr)**	**Male Sprague Dawley Rats Plasma CT-101S**				**Average**	**SD**	**RSD**
**0.25**	575	556	580		570	13	2%
**0.5**	690	855	854		800	95	12%
**1**	712	1,060	1,430		1,067	359	34%
**2**	589	1,160	1,300		1,016	377	37%
**4**	386	773	749		636	217	34%
**8**	135	386	337		286	133	47%
**24**	94	238	205		179	75	42%
**Average RSD**							**30%**
	**CD-1 Mice Plasma CT-101S**				**Average**	**SD**	**RSD**
**0.25**	1,790	1,320	640	1,340	1,273	474	37%
**0.5**	2,590	2,010	1,510	1,920	2,008	445	22%
**1**	3,570	2,090	2,170	2,080	2,478	729	29%
**2**	1,850	1,570	2,570	1,730	1,930	442	23%
**4**	1,080	629	2,060	1,160	1,232	599	49%
**8**	287	186	788	424	421	263	62%
**24**	22.8	18.3	48.5	17.8	27	15	54%
**Average RSD**							**40%**
	**Townes Mice Plasma CT-101S**				**Average**	**SD**	**RSD**
**0.5**	1,860	2,620	1,560		2,013	546	27%
**1**	1,560	1,650	2,040		1,750	255	15%
**3**	4,290	676	742		1,903	2,068	109%
**6**	1,130	2,600	261		1,330	1,182	89%
**12**	922	107	ISV		515	ND	ND
**24**	ISV	40.5	53.2		47	ND	ND
**Average RSD**							**60%**

*All reported concentrations in **ng/mL**
Abbreviations: BLQ, below limit of quantitation; **ISV,** insufficient volume to perform assay; **ND,** not done

**S5 Table. Summary of pharmacokinetic data for CT-101 in mice and rats.**								
**Animal Model**	**CT-101 Analogue**	**Parameter**	**Cmax**	**Tmax**	**AUClast**	**Tlast**	**$T_{1/2}$**	**Number of Data Points**
			**(ng/mL)**	**(hr)**	**(hr*ng/mL)**	**(hr)**	**(hr)**	**(n)**
Male CD-1 Mice	Thiol	Mean	3,890	2.2	12,000	8	5.3	n = 4
	Thiol	SD	1,090	3.9	8,110		5.5	
	Thiol	%RSD	28	177	68		104	
	Dimer	Mean	95	1.0	163	2	1.0	n = 4
	Dimer	SD	47	0.7	197		0.4	
	Dimer	%RSD	50	70.7	121		43	
Male CD-1 Mice	Thiol	Mean	2,580	1.3	13,900	24	3.8	n = 4
	Thiol	SD	700	0.5	4,890		0.3	
	Thiol	%RSD	27	40	35		8	
	Dimer	Mean						ND
Townes Mice	Thiol	Mean	2,010	0.5	18,800	24	3.7	n = 3
	Dimer	Mean	635	1.0	3,020	0.5	3.1	n = 2
Male Sprague Dawley rats	Thiol	Mean	1,100	1.3	7,610	24	7.1	n = 3
	Thiol	SD	363	0.6	2,760		0.3	
	Thiol	%RSD	33	43	36		5	
	Dimer	Mean						ND

Abbreviations: **C_{max}**, maximum concentration; **T_{max}**, time of maximum concentration; **AUC_{last}**, area under the curve; **T_{last}**, final time point; **$T_{1/2}$**, half-life; **RSD,** relative standard deviation; **ND**, not done.

Supporting Figures

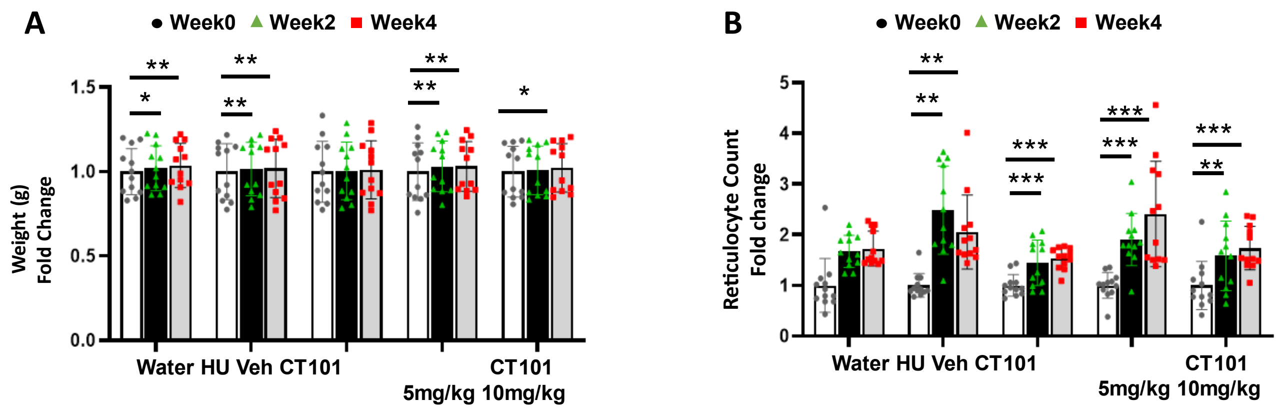

S1 Figure. Treatment of β-YAC mice with CT-101. To establish the dose of CT-101 that induces fetal hemoglobin expression without blood toxicities, mice were treated with CT-101 5mg/kg and 10 mg/kg, three days a week (M, W, and F) for four weeks. For all graphs, n=12 mice in each treatment group. **A)** The weights of mice were recorded at weeks 0, 2, and 4 to monitor eating habits. **B)** As another measure of blood toxicity, the reticulocyte counts were done by flow cytometry with acridine orange at weeks 0, 2, and 4. Data are shown as the mean ± standard error of the mean; $p<0.05$ is considered significant. Symbols: *=$p<0.05$; **=$p<0.01$; ***=$p<0.001$.

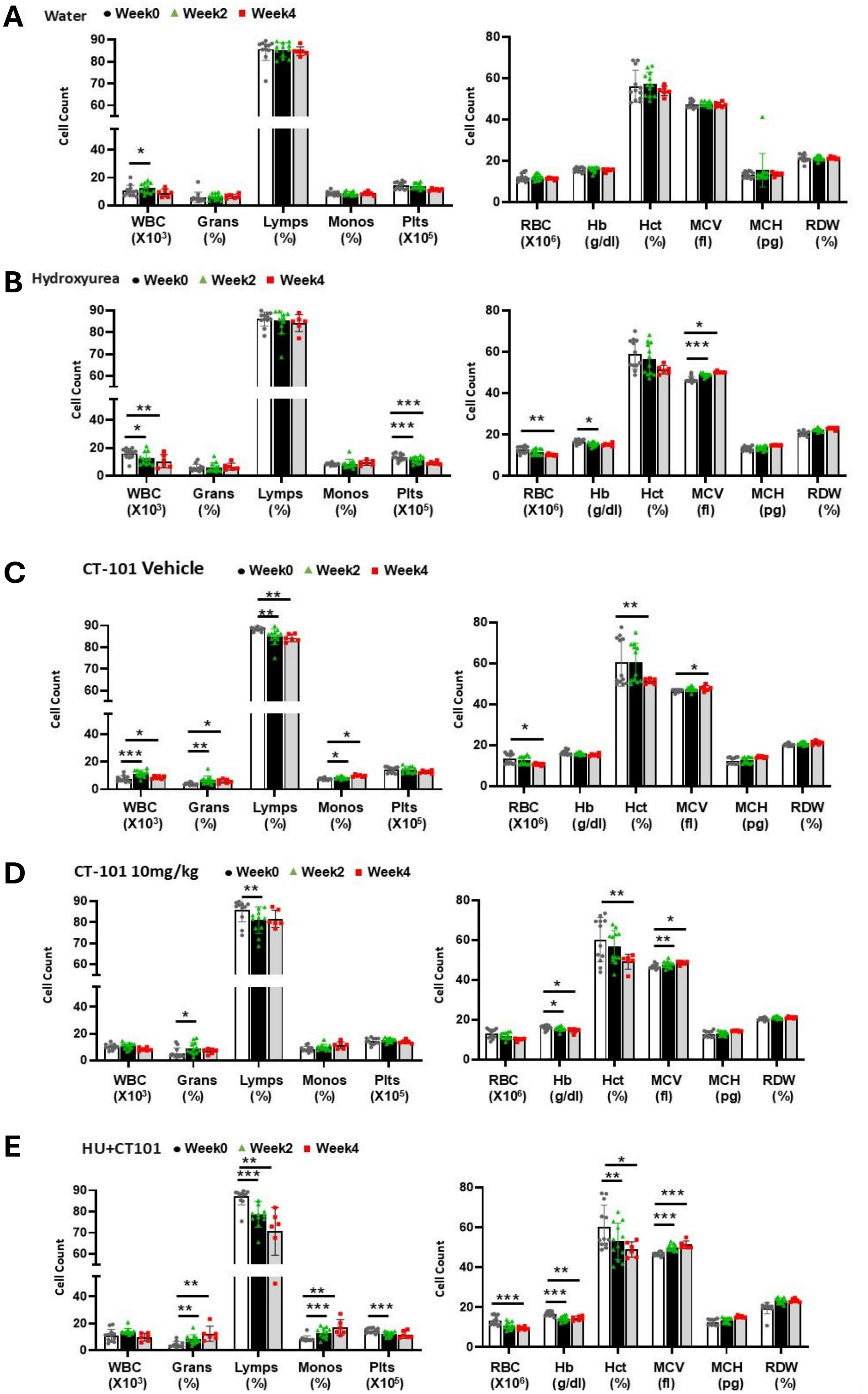

S2 Figure. Complete blood cell counts and differential after CT-101 treatment in β-YAC mice. Study 2 was performed to determine whether combined CT-101 and HU treatment enhance fetal hemoglobin induction in an additive manner. The study design remained the same as Study 1 except for the CT-101/HU group. The effects in the peripheral blood are shown. **A-E)** The data are shown for water, hydroxyurea, vehicle, CT-101, and HU+CT101 combined. Abbreviations: WBC, white blood cells; Grans, granulocytes; Lymps, lymphocytes; Mono, monocytes; Plts, platelets; RBC, red blood cells; Hb, hemoglobin; Hct, hematocrit; MCV, mean corpuscular volume; MCH, mean corpuscular hemoglobin; RDW, red cell distribution width. Data are shown as the mean ± standard error of the mean; $p<0.05$ is considered significant. Symbols: *=$p<0.05$; **=$p<0.01$; ***=$p<0.001$.

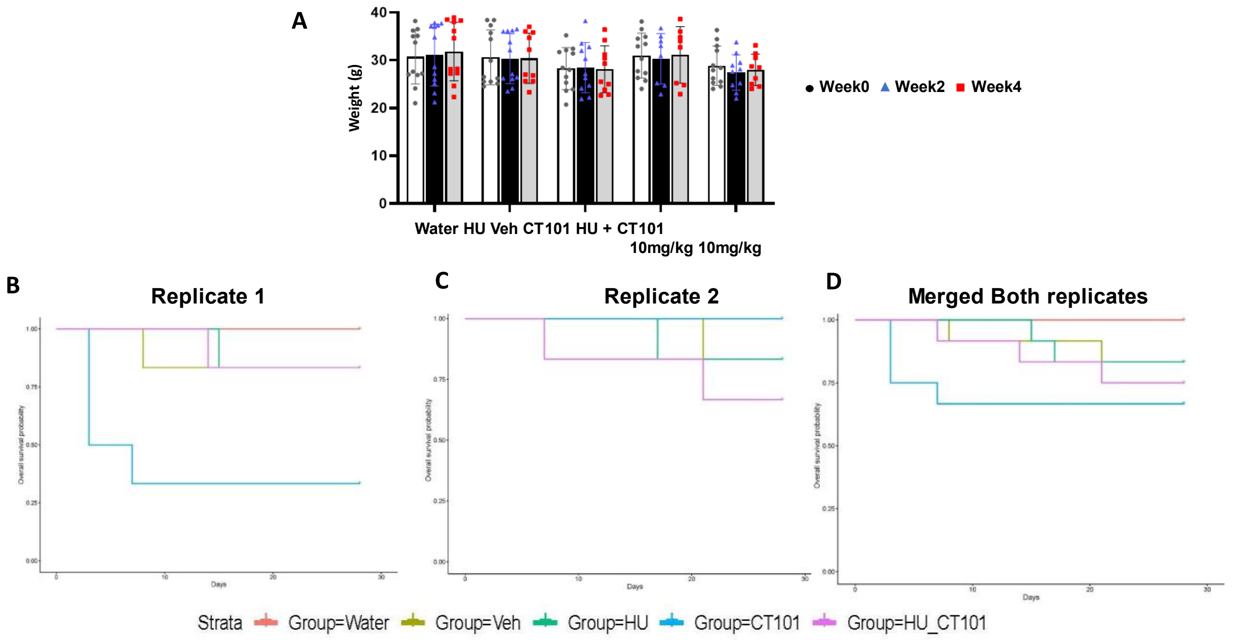

S3 Figure. Study 3 survival curves of sickle cell disease mice (SCD) treated with CT-101. Study 3 was performed in SCD mice to determine whether CT-101 induces fetal hemoglobin under oxidative stress conditions. The study design remained the same as established for β-YAC mice. **A)** Five treatment groups were conducted, including CT-101 10mg/kg, HU 100mg/kg, and combined HU/CT-101 along with water and FORM-3 (Veh) control treatments. Mice were weighed at weeks 0, 2, and 4. Data are shown as the mean $\pm$ standard error of the mean; $p<0.05$ is considered significant. **B)** Study 3, Replicate 1, survival curve for the different treatment groups including water (n=12), HU (n=10), veh (n=10), CT-101 (n=8) and HU/CT101 (n=9). **C)** Study 3, Replicate 2 was completed with the same experiment; n=6 mice per treatment group. **D)** Summary of the merged data for both treatment replicates.

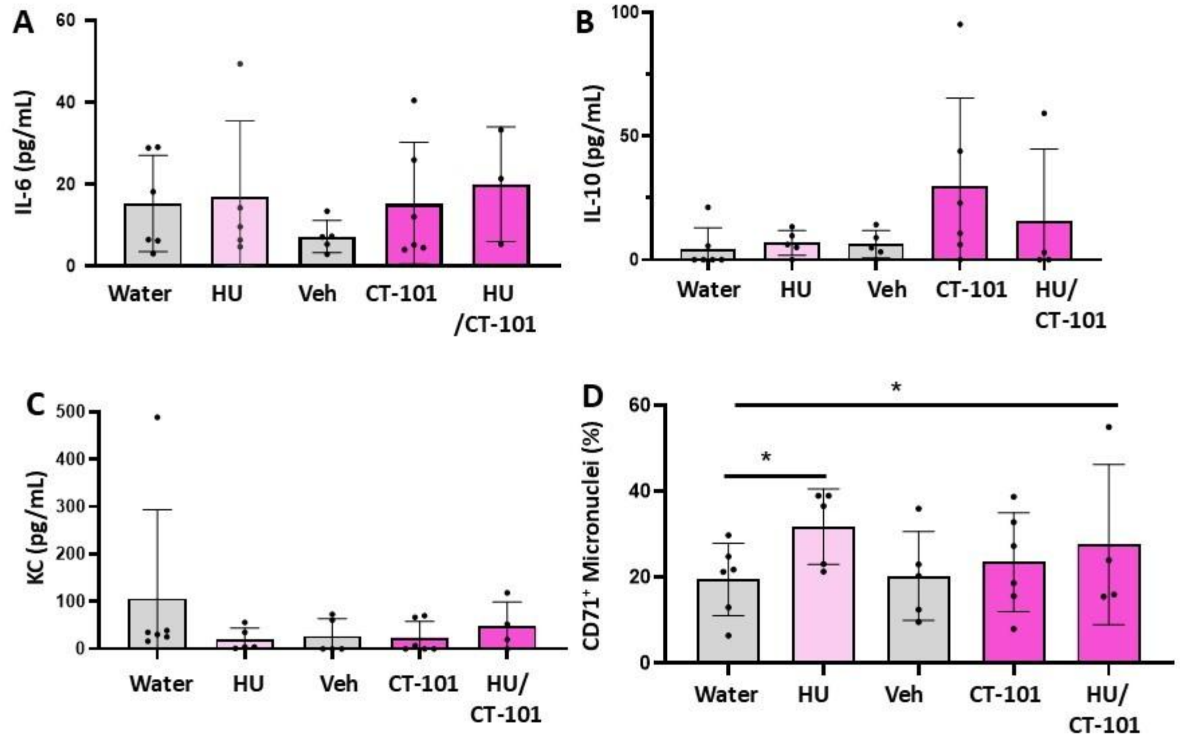

S4 Figure. CT-101 does not increase micronuclei in red blood cells. Plasma was collected from SCD mouse blood obtained by cardiac puncture at the end of the treatment period for plasma cytokine analysis using a multiplex system. **A)** The quantitative data for interleukin-6 (IL-6) levels for the different treatment groups is shown. **B)** The quantitative data for interleukin-10 (IL-10) levels of the different treatment groups is shown. **C)** The quantitative data for KC levels is shown. KC is the mouse cytokine analogous to human interleukin 8 (neutrophil attractant). **D)** Shown are the levels of micronuclei to determine whether CT-101 mediates carcinogenic effects (See Materials and Methods). The graph shows the different treatment group micronuclei counts. Symbols: *=p<0.05, **=p,0.01, ***=p<0.001.

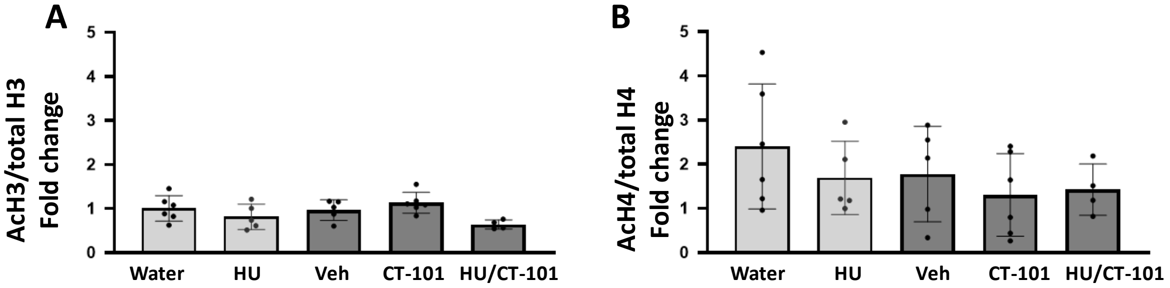

S5 Figure. Histone acetylation levels in the spleen of SCD mice. At the end of treatment, SCD mice were sacrificed, and spleens were harvested for Western blot analysis as previously published by our group [29]. For each group, the number of mice is water (n=6), HU (n=5), Veh (n=5), CT-101 (n=6), and HU/CT-101 (n=4). Protein was isolated from spleen tissue, quantified by Bradford method, and used for Western blot analysis (see Material and Methods). There were not statistically significance differences between water and HU or Veh and CT-101 and HU/CT-101 treatments.
