# Supplementary material for: The histone deacetylase inhibitor CT-101 flips the switch to fetal hemoglobin expression in sickle cell disease mice
Source: PLoS One. 2025 May 13;20(5):e0323550. doi: 10.1371/journal.pone.0323550 (PMC12074596; doi:10.1371/journal.pone.0323550)
Supplement: S1 Raw images — (PDF) [file pone.0323550.s002.pdf]

Raw data for histone acetylation studies of spleens from sickle cell disease mice.

Group		H3	AcH3	AcH3/H3
	Mouse ID			
**Water**	7434M	2081	3030	1.456030754
	7505M	3029	2680	0.884780456
	7443M	2309	2673	1.157644002
	7522F	3675	2300	0.62585034
	7524F	2543	2743	1.078647267
	7556F	3776	3106	0.822563559
**HU**	7614	3719	1916	0.515192256
	7615	3148	1930	0.613087675
	7616	2458	2981	1.212774614
	7531F	3424	3441	1.004964953
	7535F	3762	2776	0.737905369
	7541F			
**Vehicle**	7549M	3732	3268	0.875669882
	7550M	2221	2566	1.155335434
	7525M	2812	3280	1.166429587
	7561F	4240	4367	1.02995283
	7617	4724	2848	0.602878916
	7618			
**CT 101**	7537M	4844	4054	0.836911643
	7538M	2131	3311	1.553730643
	7551M	2461	2720	1.105241772
	7564F	3022	3107	1.028127068
	7566F	2800	3294	1.176428571
	7567F	2752	2993	1.087572674
**CT/HU**	7544M	3560	2438	0.684831461
	7545M	1924	1044	0.542619543
	7547M	2883	1620	0.561914672
	7621	1995	1520	0.761904762
	7619			
	7620			

Yellow box: data not acquired

Group		H4	AcH4	AcH4/H4
	Mouse ID			
**Water**	7434M	2760	3371	1.221376812
	7505M	3268	5401	1.652692778
	7443M	3507	3368	0.960364984
	7522F	1372	6216	4.530612245
	7524F	669	2402	3.590433483
	7556F	1169	2872	2.456800684
**HU**	7614	3874	4574	1.180691791
	7615	3120	6584	2.11025641
	7616	4580	4571	0.998034934
	7531F	1773	5239	2.954878737
	7535F	1567	1899	1.211869815
	7541F			
**Vehicle**	7549M	4935	4845	0.981762918
	7550M	3564	7622	2.138608305
	7525M	2420	6985	2.886363636
	7561F	2666	6804	2.552138035
	7617	3486	1172	0.336201951
	7618			
**CT 101**	7537M	4801	3825	0.796709019
	7538M	2378	5428	2.282590412
	7551M	3616	5945	1.644081858
	7564F	1999	4813	2.407703852
	7566F	3050	1335	0.437704918
	7567F	2774	729	0.262797404
**CT/HU**	7544M	2661	2180	0.819240887
	7545M	2915	3444	1.181475129
	7547M	2773	4196	1.51316264
	7621	1493	3263	2.185532485
	7619			
	7620			

AcH3/H3		
Group	Mean	SEM (Raw)
Water	1.00425	0.1188332
HU	0.81678	0.1286259
Vehicle	0.96605	0.104911
CT 101	1.13134	0.0966755
CT/HU	0.63782	0.0519871

AcH4/H4		
Group	Mean	SEM (Raw)
Water	2.4020468	0.576773
HU	1.6911463	0.370361
Vehicle	1.779015	0.4832478
CT 101	1.3052646	0.3822461
CT/HU	1.4248528	0.2904638

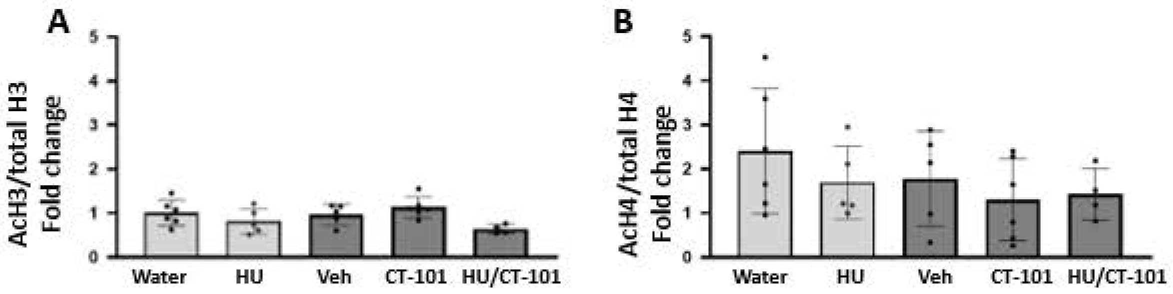

Figure S5. Histone acetylation levels in the spleen of SCD mice. At the end of treatment, SCD mice were sacrificed, and spleens were harvested for Western blot analysis as previously published by our group [29]. For each group, the number of mice is water (n=6), HU (n=5), Veh (n=5), CT-101 (n=6), and HU/CT-101 (n=4). Protein was isolated from spleen tissue, quantified by Bradford method, and used for Western blot analysis (see Material and Methods). There were not statistically significance differences between water and HU or Veh and CT-101 and HU/CT-101 treatments.

Original Western blot gels

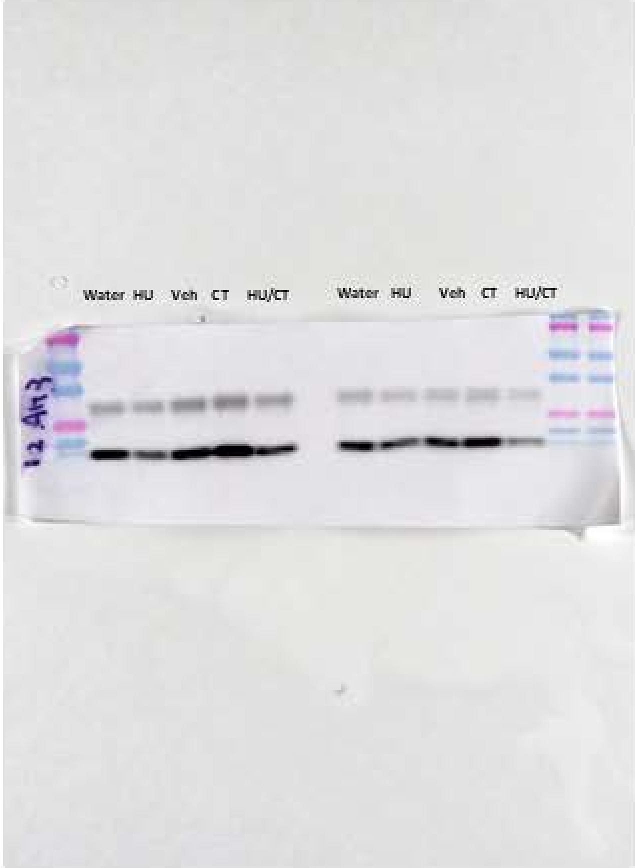

Acetylated histone H3

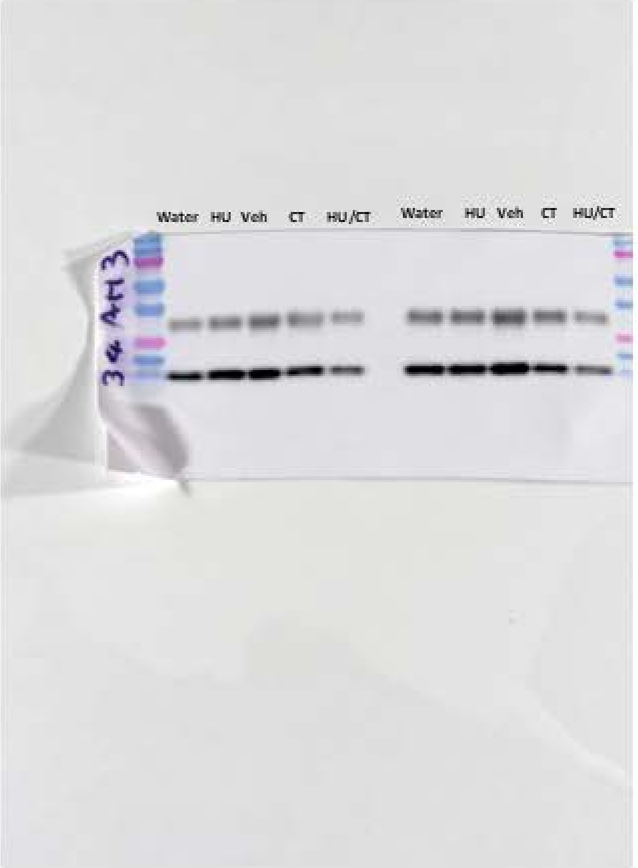

Acetylated histone H3

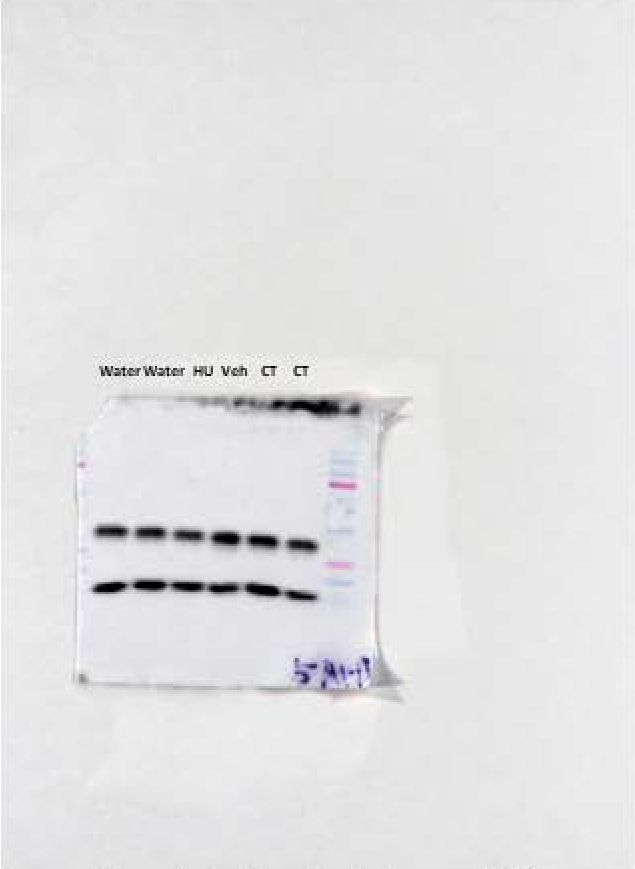

Acetylated histone H3

Original Western blot gels

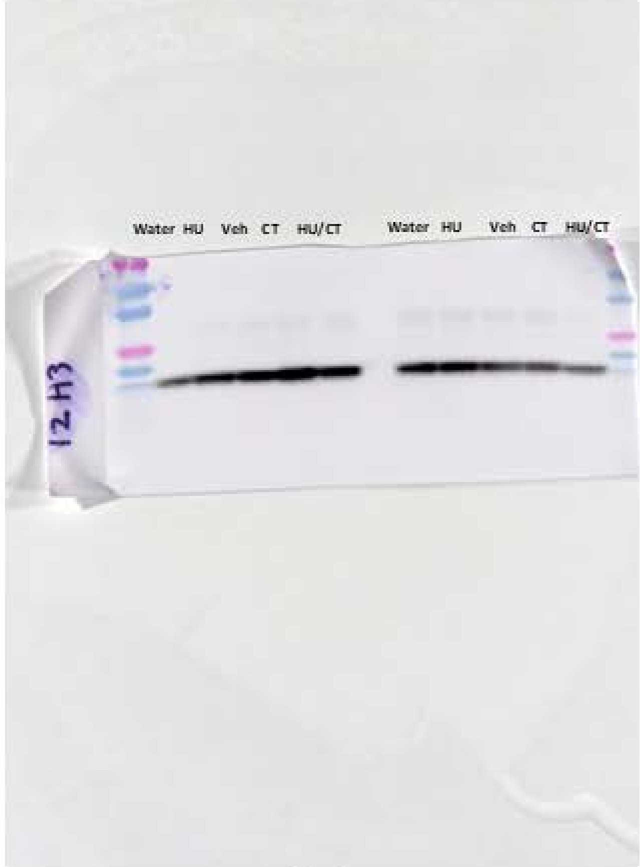

Total histone H3

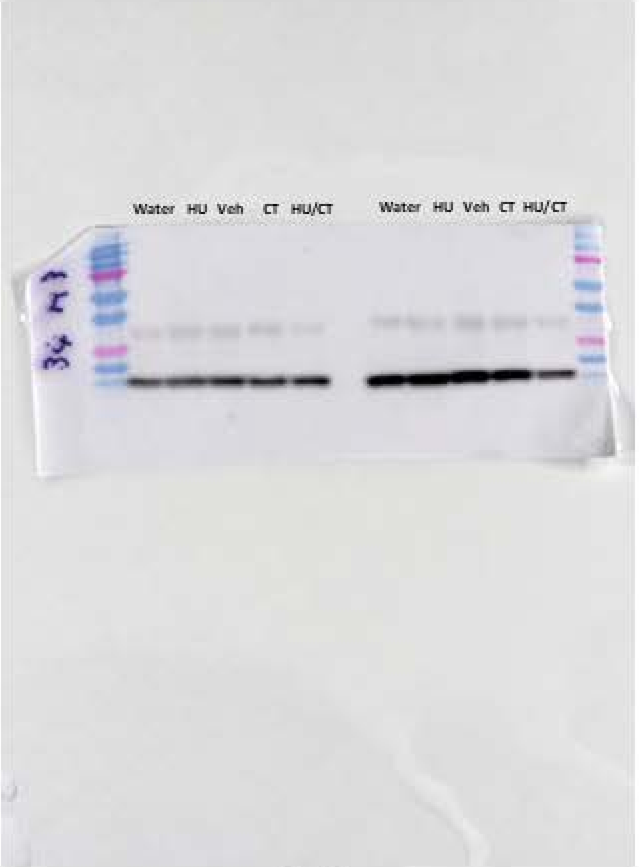

Total histone H3

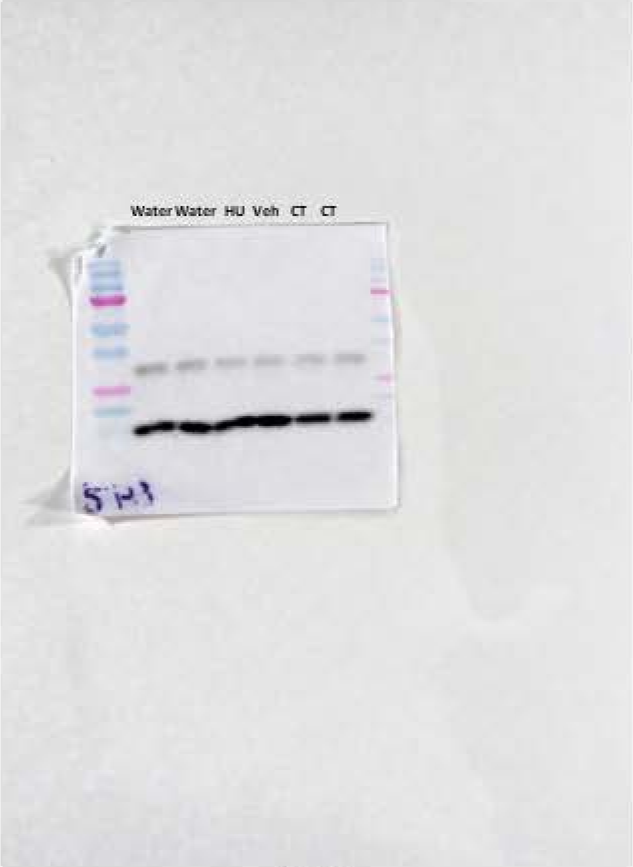

Total histone H3

Original Western blot gels

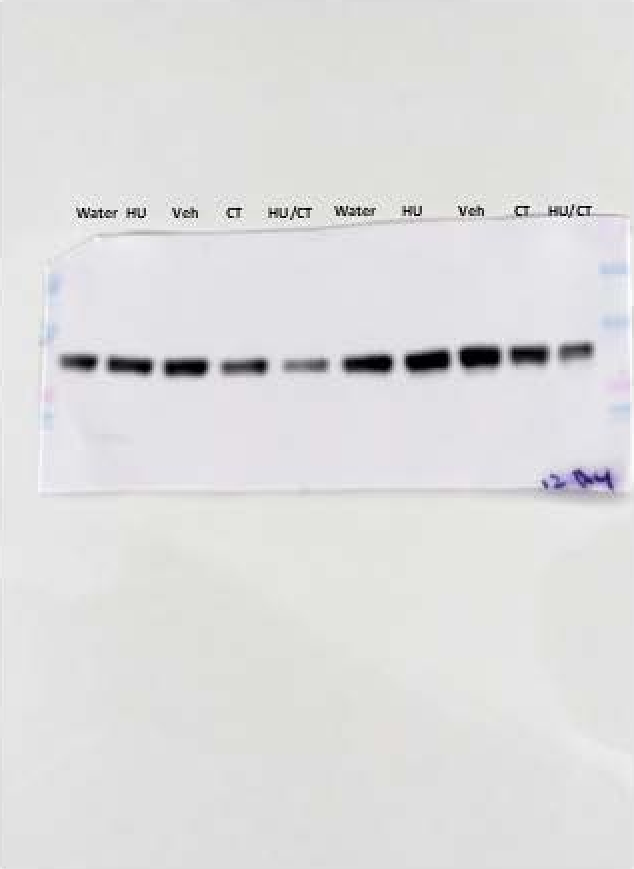

Acetylated histone H4

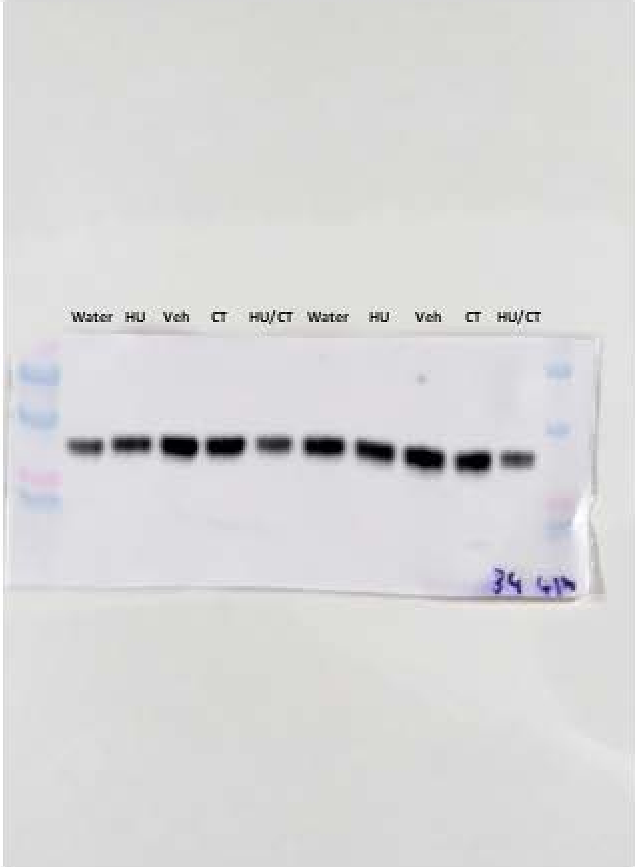

Acetylated histone H4

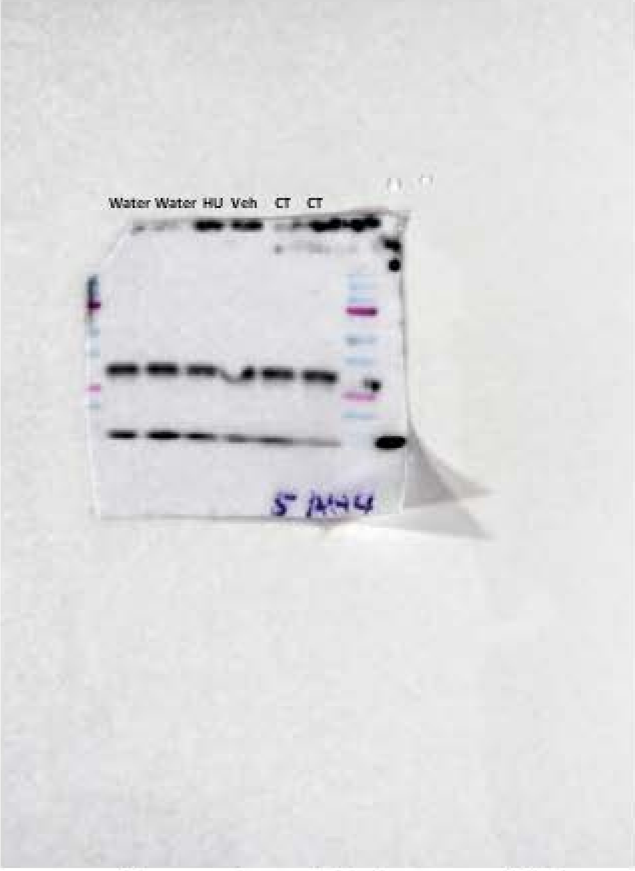

Acetylated histone H4

Original Western blot gels

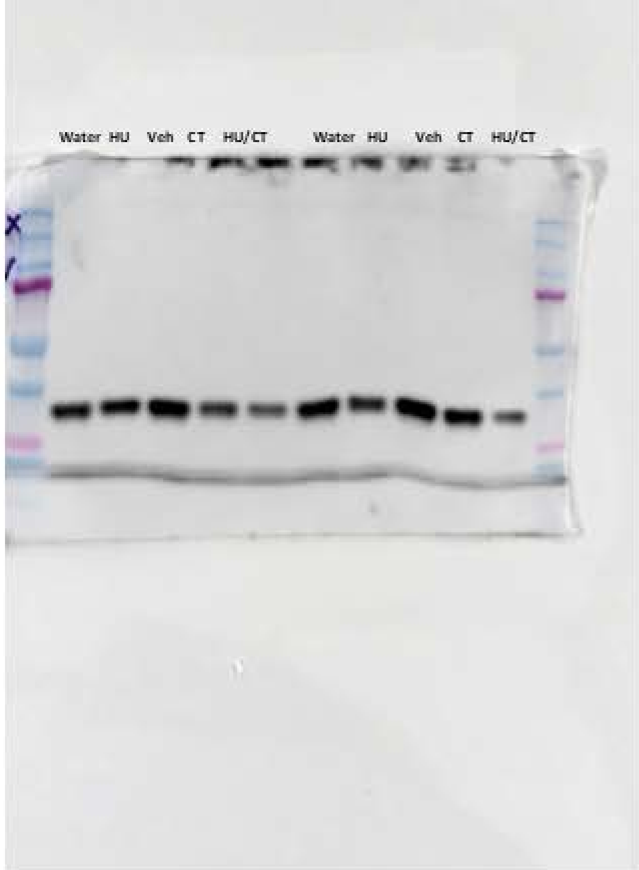

Total histone H4

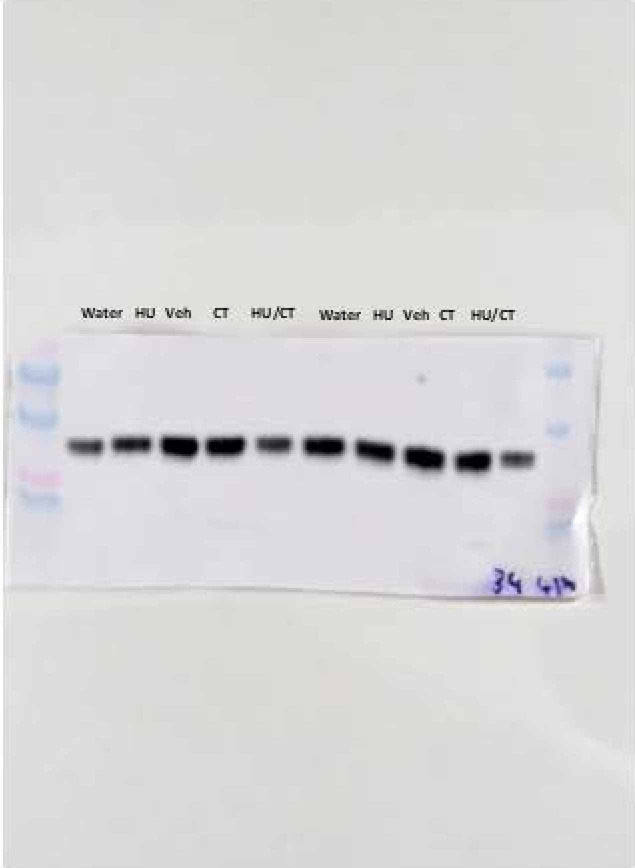

Total histone H4

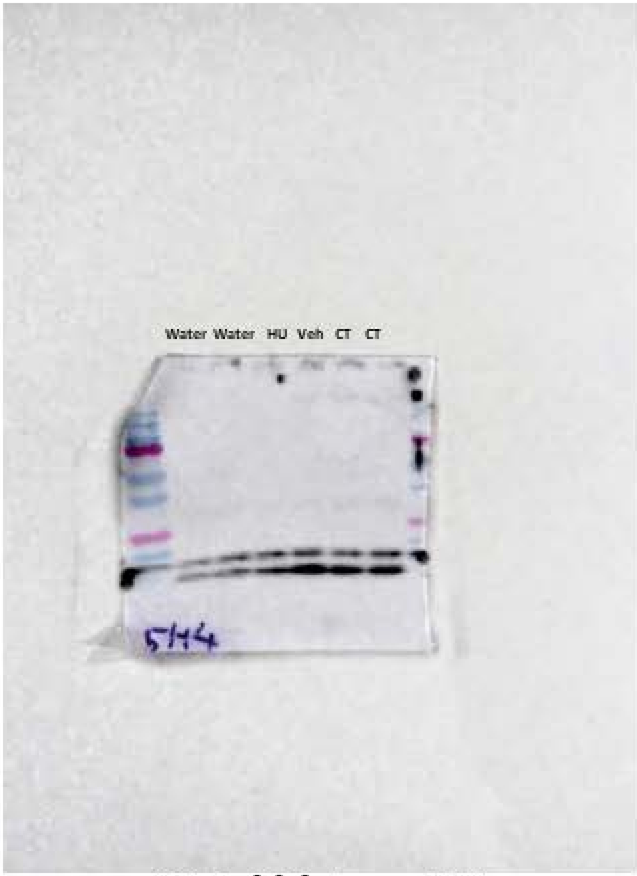

Total histone H4
